# Evolution of Nova-Dependent Splicing Regulation in the Brain

**DOI:** 10.1371/journal.pgen.0030173

**Published:** 2007-10-12

**Authors:** Nejc Jelen, Jernej Ule, Marko Živin, Robert B Darnell

**Affiliations:** 1 Laboratory of Molecular Neuro-Oncology, The Rockefeller University, New York, New York, United States of America; 2 Howard Hughes Medical Institute, The Rockefeller University, New York, New York, United States of America; 3 Division of Structural Studies, MRC Laboratory of Molecular Biology, Cambridge, United Kingdom; 4 Brain Research Laboratory, Institute of Pathophysiology, University of Ljubljana, Ljubljana, Slovenia; Tel Aviv University, Israel

## Abstract

A large number of alternative exons are spliced with tissue-specific patterns, but little is known about how such patterns have evolved. Here, we study the conservation of the neuron-specific splicing factors Nova1 and Nova2 and of the alternatively spliced exons they regulate in mouse brain. Whereas Nova RNA binding domains are 94% identical across vertebrate species, Nova-dependent splicing silencer and enhancer elements (YCAY clusters) show much greater divergence, as less than 50% of mouse YCAY clusters are conserved at orthologous positions in the zebrafish genome. To study the relation between the evolution of tissue-specific splicing and YCAY clusters, we compared the brain-specific splicing of Nova-regulated exons in zebrafish, chicken, and mouse. The presence of YCAY clusters in lower vertebrates invariably predicted conservation of brain-specific splicing across species, whereas their absence in lower vertebrates correlated with a loss of alternative splicing. We hypothesize that evolution of Nova-regulated splicing in higher vertebrates proceeds mainly through changes in *cis*-acting elements, that tissue-specific splicing might in some cases evolve in a single step corresponding to evolution of a YCAY cluster, and that the conservation level of YCAY clusters relates to the functions encoded by the regulated RNAs.

## Author Summary

Alternative splicing generates different mRNA isoforms from a single gene and thus increases the number of proteins a cell can produce. This is particularly important in the brain, which possesses a number of brain-specific splicing factors. In this study, we have looked at evolution of brain-specific splicing regulation by one such factor, Nova. Previous studies have identified ∼100 alternative exons that are regulated by Nova in mouse brain. We find that the Nova protein sequence changed little during vertebrate evolution from fish to human, whereas the RNA targets themselves have evolved significantly. Interestingly, the presence of conserved Nova binding elements in an RNA transcript in most cases correlates with conservation of brain-specific splicing. In addition, the evolution of Nova-dependent splicing relates to the functions encoded by the target RNAs, such that Nova-regulated splicing of RNAs encoding core roles such as synaptic adhesion, ion channel, and cytoskeletal proteins is on average more conserved than splicing of the RNAs encoding regulatory roles, such as transmembrane receptor and signal transduction proteins.

## Introduction

Alternative splicing is believed to be one of the major mechanisms by which proteome diversity is generated in multicelullar organisms [1,2]. Initial estimates that 5% of human genes are alternatively spliced [3] have recently been revised, such that it is now believed that 40%–60% of human genes are alternatively spliced [4,5]. A large number of these alternative exons are spliced with brain-specific patterns, as shown by microarray studies [6,7]. Such tissue-specific splicing patterns require interactions between defined *cis*-acting sequences present in the vicinity of the alternative exons and *trans*-acting regulatory factors [8,9]. It is still an open question to what extent mutations in *cis*-acting sequences or in genes encoding *trans*-acting regulatory factors have contributed to the evolution of splicing regulation [10,11], or to the development of neurologic disease [12].

Analysis of the evolutionary conservation of alternative exons can provide evidence of the regulation and functional significance of the exons. In addition, since alternatively spliced exons and flanking intronic regions are generally more conserved than constitutive exons, analysis of evolutionary conservation can be used to identify de novo alternative exons and regulatory sequences [13]. Interestingly, analysis of alternative exon conservation between human and mouse transcriptomes revealed a relationship to their inclusion level: major exons, included at over 50%, were found to be highly conserved (98%), whereas minor exons, included below 50%, were poorly conserved (26%) [14,15]. However, if a minor alternative exon shows a tissue-specific splicing pattern, its conservation between human and mouse rises to the level of major exons [16]. In general, exons with tissue-specific splicing patterns have increased conservation and frame preservation relative to other alternative exons [16,17].

Nova is a brain-specific splicing factor, first identified as an antigen in a neurologic disorder termed paraneoplastic opsoclonus-myoclonus ataxia [18]. Nova binds to clusters of tetranucleotide YCAY motifs (YCAY clusters), which are often present in the vicinity of Nova-regulated alternative exons [19–22]. When these are positioned within 200 nucleotides of the splice sites, their position predicts whether Nova functions to inhibit or enhance alternative exon inclusion, following the rules of an RNA map. So far, biochemical studies and analysis of mouse genomic sequence have found 76 different YCAY clusters located within Nova-regulated pre-mRNAs [6,20–24]. Fifty-four of these clusters are located in genes that are expressed both in brain and liver. This allowed us to analyze the splicing pattern of Nova-regulated exons from brain and liver mRNA of different vertebrates and relate it to the conservation of YCAY clusters. We found that a brain-specific splicing pattern was conserved across species in all (24/24) tested cases where YCAY clusters were conserved. We also found seven cases lacking the brain-specific splicing pattern, which correlated with the absence of detectable YCAY clusters in the corresponding pre-mRNAs. Interestingly, in all of these cases, we also observed a loss of alternative splicing (i.e., a single isoform detected in brain and liver). This suggests that some RNAs might have acquired brain-specific splicing regulation in a single step by addition of the YCAY cluster, without the need for preliminary mutations that would create an alternative exon.

## Results

**Figure 1:**
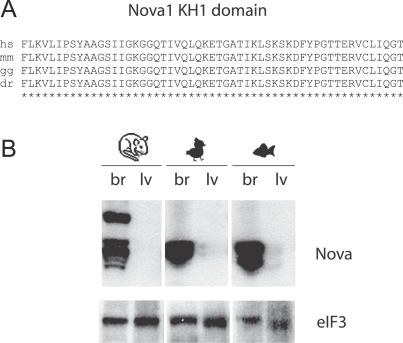
Analysis of Nova Protein in the Brain and Liver of Different Vertebrate Species (A) Protein alignment of the 100% conserved region of the Nova1 KH1 domain. (B) Immunoblot analysis of brain and liver extract from mouse, chicken, and zebrafish using polyclonal Nova and the eIF3a loading control antibody. Protein (50 μg) was loaded in each lane. Mouse brain contains an additional slower migrating Nova band that is not detected in chick and zebrafish brain (but is present in human brain). In another study, we cloned this slower migrating band and found that it binds to similar YCAY clusters and regulates the same exons as the previously cloned Nova1 and Nova2 isoforms (unpublished data). The abbreviations for the following species are used: *H. sapiens* (hs), M. musculus (mm), *G. gallus* (gg), and D. rerio (dr).

Recently, an RNA map was constructed to relate Nova-dependent splicing regulation in brain to the position of its binding site (YCAY cluster) on the pre-mRNA [24]. This RNA map defines how YCAY clusters at five major positions on pre-mRNA direct the action of Nova on the splicing of neighboring alternative exons. At two such positions, YCAY clusters act as splicing enhancers and at three positions as splicing silencers. Seventy-six such YCAY clusters were found within Nova-regulated pre-mRNAs, providing a tool to relate the conservation of YCAY clusters to the evolution of brain-specific splicing. Nova proteins show high conservation between different vertebrate species, with the amino acid sequence of Nova1 and Nova2 KH domains 94% identical between zebrafish and mouse orthologues (Figures 1A and S1). Moreover, we found that both Nova1 and Nova2 bind to similar YCAY clusters in vivo, using the cross-linking and immunoprecipitation (CLIP) method ([22,25], unpublished data). We also confirmed that Nova expression is restricted to the brain in chicken and zebrafish (Figure 1B).

**Figure 2:**
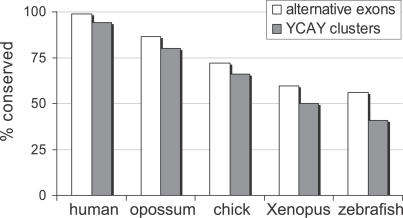
Conservation of Mouse YCAY Clusters and Nova-Regulated Alternative Exons in Different Vertebrates We analyzed conservation of 49 mouse YCAY clusters that were predicted by microarray. We observed a high degree of conservation of YCAY clusters across species, with 94% conservation in human and 41% conservation in zebrafish. We also analyzed genomic conservation of 88 mouse Nova-regulated alternative exons in vertebrates, based on cDNA/EST data, genomic alignment, and sequencing. The results were similar to conservation of YCAY clusters, with 56% of exons conserved in zebrafish and 99% in human. Because cDNA/EST data is not complete and may contain errors, we may have overestimated or underestimated the level of alternative exon conservation, but in general our sequencing results agreed very well with cDNA/EST or genomic prediction.

Given the highly conserved nature of the Nova proteins, we hypothesized that the main driving force in the evolution of Nova-dependent splicing regulation in vertebrate species might be changes in YCAY clusters within pre-mRNAs. We analyzed YCAY clusters in the pre-mRNAs containing 49 Nova-regulated exons that were previously identified by splicing microarray analysis of Nova knockout mouse brain [6]. Using the same YCAY cluster score algorithm as in the previous study [24], we found that 30% of YCAY clusters were conserved at orthologous zebrafish positions (Table S1A). This algorithm, which detects clusters with a maximum distance of three nucleotides between YCAY motifs [24], was written to be very stringent, since limiting the analysis to the highest affinity Nova-binding sites was important for precision in defining the original RNA map. However, previous analyses of minimal functional Nova binding site and of in vivo Nova–RNA interactions via the CLIP method [19–23] have shown that in some cases more dispersed YCAY clusters can be functional, even though they may bind Nova with a lower affinity. Indeed, manual analysis of sequence alignments (Figure S2) shows that the orthologous sequences often contain more dispersed YCAY clusters than the mouse genome. To be able to take these YCAY clusters into account, we modified the algorithm for the purpose of this study to detect clusters with distance of up to nine nucleotides between YCAY motifs. Analysis of gene sequences orthologous to mouse showed that 41% of the pre-mRNAs contain a conserved YCAY cluster in zebrafish, 66% in chicken, 82% in opossum, and 94% in the human genome (Figure 2; Table S1B).

To assess whether the presence of YCAY clusters can predict conservation of brain-specific splicing in vertebrates as an independent variable, we used RT-PCR to analyze brain-specific splicing of orthologous exons in zebrafish and chicken. Seventy-six different YCAY clusters located within the Nova-regulated pre-mRNAs were assigned a cluster score ≥0.6 by analysis of mouse genomic sequence using the modified YCAY cluster score algorithm [24] (Table S2 and Materials and Methods). Fifty-four (54/76) of these clusters are located in genes that are expressed both in brain and liver (Table S3). We designed PCR primers for these genes using cDNA and EST data or alignment of orthologous alternative exons and flanking constitutive exons. We were able to design PCR primers and obtain RT-PCR data for 25 chicken orthologous exons and 14 zebrafish orthologous exons.

**Figure 3:**
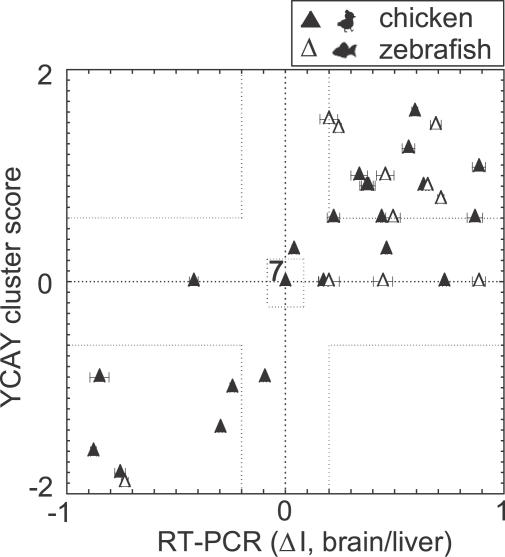
Comparison of YCAY Cluster Score to Brain-Specific Splicing in Chicken and Zebrafish The graph shows relative exon inclusion levels against calculated YCAY cluster scores. Dashed lines indicate cut-off values for conserved YCAY clusters (score > 0.6) and |ΔI| (20% change in exon inclusion between brain and liver). Data for chicken are marked with full triangles and data for zebrafish with empty triangles. From 24 cases in which exons had conserved YCAY clusters, 17 were splicing enhancers (upper right part of the graph, denoted with dotted line) and seven were splicing silencers (lower left part of the graph, denoted with dotted line). Note that the direction of splicing always correlates with the type of YCAY cluster (enhancer versus silencer) present; therefore, the upper left and the lower right part of the graph are empty. From 15 cases where YCAY clusters were not conserved, seven showed no alternative splicing (center of the graph, denoted with dotted square). Overall, we observed 31 cases where the presence of YCAY clusters matched with splicing patterns in vivo. The exceptions that did not match with our prediction were eight cases in which brain-specific splicing was present in the absence of conserved YCAY clusters. The data drawn in this graph are also shown in Table 1.

RT-PCR analysis showed a striking correlation between the presence of YCAY clusters and the conservation of brain-specific splicing. Together, we tested 39 independent splicing events (25 in chicken and 14 in zebrafish). In 24 cases, the YCAY cluster was conserved (YCAY cluster score ≥0.6 in the orthologous sequence), and RT-PCR analysis showed at least 20% change in exon inclusion between brain and liver in 23/24 cases (|ΔI|>0.20, p<0.05, Figure 3; Table 1; Figure S2, where ΔI is used as defined previously [6]). In one case (chicken efna5 gene), the difference in exon inclusion between brain and liver was smaller, but still significant (ΔI = −0.09 and *p* = 0.008). Position of the YCAY cluster in all cases correctly predicted the direction of alternative splicing, following the rules of RNA map [24]. Seventeen splicing enhancers predicted higher exon inclusion in the brain, whereas seven splicing silencers predicted lower exon inclusion in the brain relative to the liver.

**Table 1:**
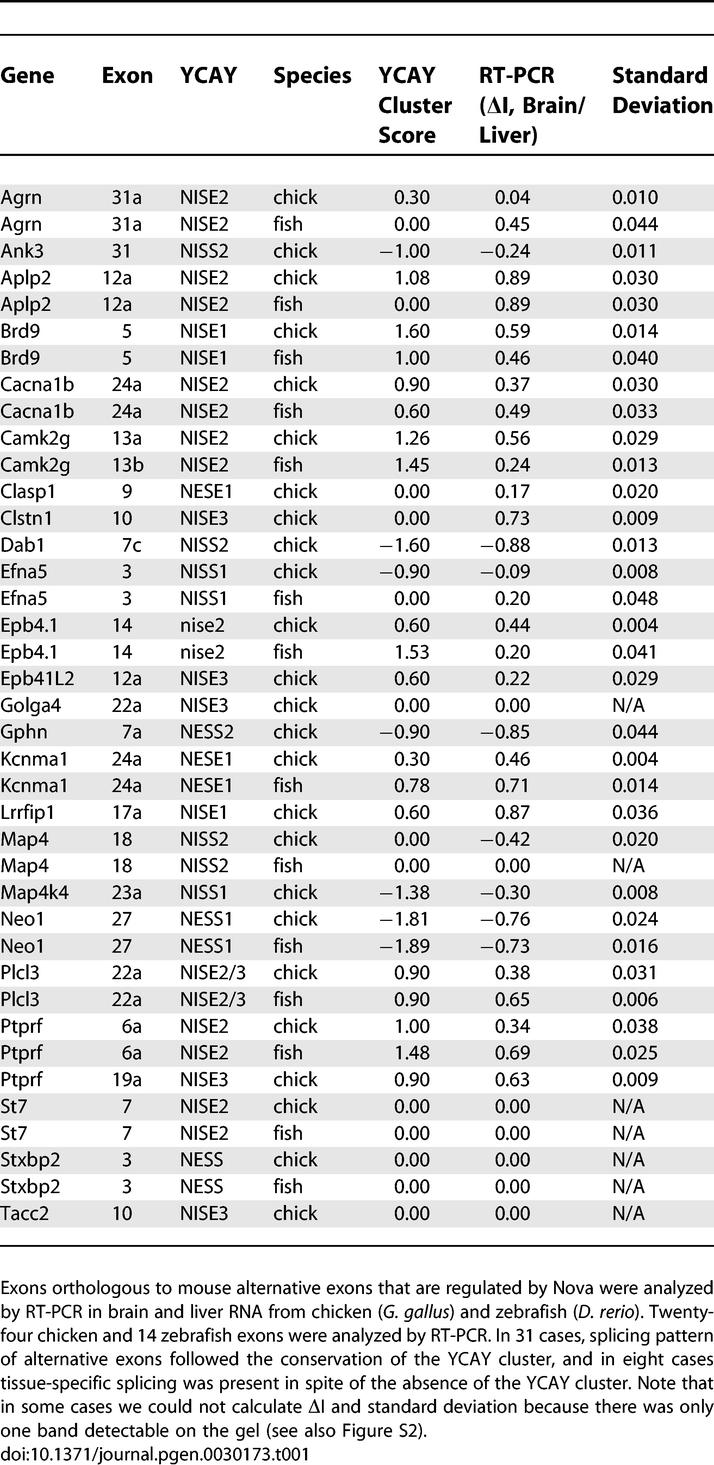
Comparison of YCAY Cluster Score to Brain-Specific Splicing in Chicken and Zebrafish

In 15 cases, YCAY clusters were not conserved (YCAY cluster score < 0.6 in the orthologous sequence). In 7/15 cases, RT-PCR detected no difference in splicing patterns of brain and liver RNA (ΔI < 0.01), and in one case (Efna5 in zebrafish) the difference in splicing pattern was the reverse of that seen in mouse. However, a significant difference in the splicing pattern of brain and liver RNA (*p* < 0.01) was detected in the remaining 7/15 cases, and in each case the change in splicing (inclusion or exclusion) was in the same direction as that seen in the mouse (Figures 3 and S2; Table 1). Thus, in 8/15 cases, the absence of YCAY cluster correlated with lack or reversal of a brain-specific splicing pattern, whereas a brain-specific splicing pattern was conserved in seven cases despite the absence of YCAY cluster in the pre-mRNA.

Taken together, we found that brain-specific splicing was conserved in all (24/24) pre-mRNAs containing YCAY clusters, but only in 47% (7/15) of pre-mRNAs lacking YCAY clusters. To address the statistical significance of this observation, we defined our problem as a comparison of these two binomial samples (first with 24/24 and second with 7/15 conservation frequency), since no previous study is available to define the expected frequency of brain-specific splicing conservation between mouse, chicken, and zebrafish. This comparison shows that the presence of a conserved YCAY cluster in pre-mRNA significantly increases the likelihood of brain-specific splicing being conserved (*p* = 0.0001, one-sided Fisher exact test).

**Figure 4:**
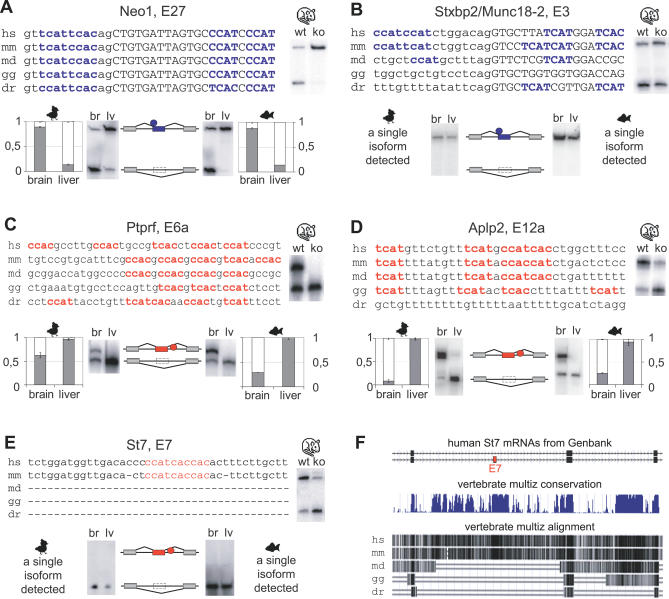
Five Examples of YCAY Cluster Conservation and RT-PCR Analysis of Associated Exons Multiple nucleotide alignment of five YCAY clusters. Introns are in lowercase, exons in uppercase, YCAY silencer motifs in bold blue, and YCAY enhancer motifs in bold red. The abbreviations for the following species are used: H. sapiens (hs), M. musculus (mm), *M. domestica* (md), G. gallus (gg), and D. rerio (dr). Next to the alignment are RT-PCR data from wild-type and Nova2 knockout brain, and below are RT-PCR data from chicken and zebrafish brain and liver and diagrams of the splicing pattern. Blue represents silencing, red enhancement by Nova, rectangles represent exons, and circles represent the position of Nova binding. (A) Nova-dependent splicing silencer in neogenin (Neo1) pre-mRNA and brain-specific splicing of exon 27 conserved. (B) Nova-dependent splicing silencer in syntaxin binding protein 2 (Stxbp2/Munc18–2) pre-mRNA and brain-specific splicing of exon 3 are not conserved. (C) Nova-dependent splicing enhancer in protein tyrosine phosphatase, receptor type, F (Ptprf) pre-mRNA and brain-specific splicing of exon 6a are conserved. (D) Nova-dependent splicing enhancer is not conserved in zebrafish amyloid beta precursor-like protein 2 (Aplp2) pre-mRNA, in contrast with conserved brain-specific splicing of exon 12a. (E) Nova-dependent splicing enhancer in suppression of tumorigenicity 7 (St7) pre-mRNA and brain-specific splicing of exon 12a are not conserved. (F) The diagram obtained from the human genome browser shows the human St7 mRNAs that either contain or exclude the alternative exon 12a. Underneath, alignment of vertebrate genomes demonstrates lack of conservation in the region containing exon 12a in the genomes of oppossum, chicken, and zebrafish.

Nova-dependent splicing silencers in *neogenin* (*Neog*, Figure 4A) and *syntaxin binding protein 2 (Stxbp2/Munc18–2*, Figure 4B) pre-mRNAs illustrate the correlations we observed between the presence of YCAY clusters and alternative splicing. In both cases, the YCAY cluster in mouse pre-mRNA spans both sides of the 3*′* splice site, so that half of the cluster is intronic, and the other half exonic. In *neogenin* pre-mRNA, the cluster and brain-specific splicing pattern of exon exclusion are conserved between zebrafish, chicken, and mouse (Figure 4A). However, Stxbp2 pre-mRNA contains a cluster of over three YCAY motifs in human and mouse, but not in opossum, chicken, and zebrafish (Figure 4B). This lack of YCAY clusters correlates with the absence of exon 3 exclusion in the brain and liver of chicken or zebrafish. This represents a case where the absence of YCAY cluster correlates with a lack of alternative splicing.

Nova-dependent splicing enhancers in *protein tyrosine kinase*, *receptor type F* (*Ptprf*, Figure 4C), and *amyloid beta (A4) precursor-like protein 2* (*Aplp2*, Figure 4D) pre-mRNAs demonstrate that brain-specific splicing is conserved at least as much as the YCAY clusters. In Ptprf pre-mRNA, the YCAY cluster and the brain-specific splicing patterns are conserved in all species (Figure 4C). In Aplp2 pre-mRNA, the YCAY cluster is conserved in chicken but not in zebrafish, whereas the brain-specific splicing pattern is conserved in all species (Figure 4D). This represents one of the seven cases where brain-specific splicing pattern is conserved in spite of the absence of detectable YCAY clusters within the pre-mRNAs (Figure 3).

A Nova-dependent splicing enhancer in *suppression of tumorigenicity* (St7, Figure 4E) pre-mRNA demonstrates a correlation between the emergence of the YCAY cluster and the alternative exon 12a. Vertebrate alignment of the St7 genomic sequence shows an absence of conservation in the region containing exon 12a in opossum, chicken, and zebrafish (Figure 4F). We detected no evidence of St7 exon 12a exon inclusion in brain and liver of chicken or zebrafish, exemplifying another case where a lack of YCAY clusters correlates with a lack of alternative splicing. This observation suggests that the YCAY cluster in St7 might have served to create the exon 12a; in the future, this hypothesis can be further analyzed using the genomic sequences of species evolutionarily intermediate to opossum and mouse.

**Figure 5:**
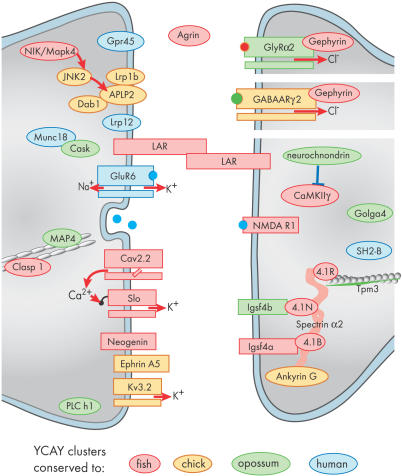
Conservation of YCAY Cluster Can Provide Insight into Evolution of Functionally Coherent Coregulated Network The diagram shows a subset of proteins encoded by Nova-regulated transcripts for which we were able to obtain orthologous sequences for analysis of YCAY cluster conservation. The color coding represents the evolutionarily most distant species from mouse that contains a conserved YCAY cluster. High YCAY cluster conservation is seen in RNAs encoding adhesion and cytoskeletal scaffold proteins, ion channels, and signaling proteins. In comparison, RNAs with lower YCAY cluster conservation (limited to mammals) most often encode neurotransmitter receptors (such as glycine [GlyRα2] and kainate [GluR6] receptors) and signaling proteins (such as neurochondrin, Lrp12, and Gpr45).

Since Nova-regulated exons encode proteins with synaptic functions [6], analysis of their splicing conservation can provide insight into the evolution of functionally coherent coregulated networks (Figure 5). We find that most RNAs with conserved YCAY clusters encode adhesion and cytoskeletal scaffold proteins, ion channels, and signaling proteins. Many of these proteins have been implicated in neuronal development, or function at the synaptic junction [26–28]. In comparison, RNAs with YCAY clusters that are conserved only in mammals encode receptors (such as glycine (GlyRα2) and kainaite (GluR6) neurotransmitter receptors) and signaling proteins (neurochondrin, Lrp12, Gpr45). It is possible that RNAs with conserved YCAY clusters more often encode proteins that are indispensable for synaptic development and function, while the RNAs with YCAY clusters present only in mammals more often encode proteins that modulate synaptic function. This hypothesis cannot yet be statistically analyzed since not enough is known about the detailed synaptic functions of each Nova-regulated gene.

## Discussion

Previous studies have shown that Nova regulates a module of alternative exons that encode a functionally coherent set of proteins. How did this relationship between an RNA-binding protein and its set of RNA targets evolve? We approached this question by analyzing evolutionary changes in Nova proteins, YCAY clusters, and splicing of corresponding alternative exons in mouse, chicken, and zebrafish. In this paper, we developed a hypothesis that evolution of Nova-regulated splicing proceeds mainly through changes in *cis*-acting elements, while Nova proteins themselves show little evolutionary change.

This is the first study to analyze the conservation of tissue-specific splicing between species other than mouse and human. While bioinformatic analysis shows alternative exon conservation between mouse and human to be roughly 75% [14], we find ∼80% (31/39) conservation of brain-specific splicing patterns between evolutionarily much more diverse mouse, chicken, and zebrafish species. This estimate might be a bit higher than the average conservation rate, since we were not able to define orthologous positions in chicken and zebrafish genomes to analyze splicing of a subset of Nova-regulated exons, due to the lower level of their genomic sequence conservation. However, the finding agrees with previous comparisons of splicing in mouse and human tissues showing that exons with tissue-specific splicing are more conserved than the rest of the alternative exons [16,17]. It will be interesting in the future to study the conservation of tissue-specific exons between such diverse species as fish and human in order to test more generally if exons with brain-specific pattern, in addition to those regulated by Nova, have particularly high conservation rates.

This study modified the original YCAY cluster algorithm by relaxing the requirement for the maximal distance between two YCAY motifs from three to nine nucleotides. The greater ability of the modified algorithm to detect conserved YCAY clusters is evident by analyzing the 31 cases where RT-PCR analysis in this study detected conservation of brain-specific splicing in chicken or zebrafish. Whereas the original more-stringent algorithm detects YCAY clusters in only 15 cases, the modified algorithm detects a conserved cluster in 24/31 cases (Table S1). A shortcoming of our current algorithm is that it analyzes the YCAY motifs as being separated by a linear RNA molecule, even though in reality YCAY motifs that appear dispersed on a linear RNA may be in relatively close proximity once the RNA assumes its in vivo structure. This may be one reason why the original study did not detect YCAY clusters in all the pre-mRNAs regulated by Nova in mouse brain, and why the current study didn't detect conserved YCAY clusters in all cases where brain-specific splicing pattern was conserved.

Of 15 pre-mRNAs in which we didn't detect a conserved YCAY cluster, seven had conserved brain-specific splicing patterns. In these instances, our algorithm may be failing to detect some types of conserved Nova-binding sites; in particular, those where RNA structure might bring multiple YCAY motifs in proximity. Alternatively, many alternative exons are regulated by multiple *cis*-acting elements [29]. For instance, splicing of alternative exon 19 in NMDA receptor1 (NR1) is regulated by Nova and several other factors including Napor, hnRNP A1, hnRNP H, and CaM kinase IV [30–32]. Thus, it is reasonable to believe that during a long evolutionary time, the importance of different *cis*-acting sites for the tissue-specific splicing pattern varies, allowing for other factors to compensate for the absence of YCAY clusters in these eight cases. We note that in our previous study [24], we demonstrated that YCAY clusters located within 200 nucleotides of splice sites predict Nova-dependent splicing regulation, but we do not know whether these clusters are the only ones required for Nova action. It is possible that in some cases Nova may be able to regulate its target exons via additional YCAY clusters located in introns further than 200 nucleotides from the splice sites, which would have been missed in this study.

We found seven cases of pre-mRNAs lacking brain-specific splicing pattern and also lacking YCAY clusters. Interestingly, in all of these cases, rather than just lacking tissue-specific splicing, we detected no evidence of alternative splicing at all in brain and liver. This observation contrasts with the currently prevailing model, which proposes that tissue-specific splicing generally evolves from the regulation of preexisting alternative exons, so that mutations in consensus 5′ and 3′ splice sites and enhancer sequences would precede subsequent mutations that lead to tissue-specific splicing [10,33]. We observed no case where an alternative exon would subsequently evolve a tissue-specific splicing pattern, although our sample size was limited. Our data suggest that tissue-specific splicing might have evolved directly, at least in some cases. This model of direct evolution of Nova-dependent alternative splicing is also supported by the observation that splice site consensus scores and exonic enhancer density in Nova-regulated exons are on average similar to constitutive exons [24].

One of the exons that obtained a Nova-dependent brain-specific splicing pattern in more recent evolution is Munc18–2 (Stxbp2) exon 3. The role of Munc18–2 in the brain is unexplored since an early study has termed it a non-neuronal Munc18–1 (Stxbp1/Sec1) homologue [34]. However, given that Nova regulates brain-specific splicing of its exon 3 in higher mammals, Munc18–2 might play a similar role in regulating neurotransmitter release as Munc18–1 [35], in a way that would contribute to brain function of higher mammals.

There is an ongoing debate regarding the functional significance of splice variants, particularly those encoded by minor exons [13,33,36–39]. Our study showed a high conservation of Nova-regulated alternative exons regardless of whether they are minor or major exons as assessed by bioinformatic analysis of EST and cDNA sequences (Tables S4 and S5). Furthermore, the functional significance of Nova-regulated exons is corroborated by conservation of reading frame; 70% (54/77) of internal cassette exons had a length multiple of three in all species in which they were present (Table S4), agreeing with previous reports that conserved alternative exons show significant increase in frame preservation [16,17,33,40].

The composite Nova binding site allows variation in the number and density of YCAY motifs to fine tune the affinity of the RNA for Nova [18,19,21]. Furthermore, the quantitative outcome of Nova binding might be refined by changing the position of YCAY clusters within the RNA [24]. However, we observed no case where YCAY cluster would change in position in a way that would qualitatively change the outcome of Nova binding (i.e., change from a splicing enhancer to silencer). We hypothesize that these features of Nova–RNA binding might allow selection in higher vertebrates to act gradually via changing the Nova binding site, rather than Nova itself, similarly to other cases where *cis*-acting elements were suggested as the main force for phenotypic evolution [11]. Furthermore, the dynamic nature of the *cis*-acting elements could help fine tune the functional coherence of the whole network of RNAs regulated by Nova (Figure 5), which were found in previous studies to primarily encode synaptic proteins [6,22,24]. In contrast, tight evolutionary fixation of Nova is essential to preserve regulation of an array of brain-specific splicing events. Interestingly, evolutionary fixation in the vertebrate lineage does not apply to all heterogeneous nuclear ribonucleoproteins, some of which were found to vary in domain structure and even in their presence/absence between mammals and fish [41,42].

The finding that a *trans*-acting factor such as Nova, which regulates multiple genes, is more conserved than the *cis*-acting sites that regulate individual genes might be anticipated, as it agrees with previous studies showing that transcriptional and micro-RNA regulators evolve more slowly than their target binding sites [11,43,44]. However, the conservation of the *cis*-acting sites observed in this study is surprisingly high, with 94% conservation of YCAY clusters between mouse and human. In contrast, biochemical analysis of *cis*-acting sites binding tissue-specific transcriptional regulators has found that 19%–59% of sites are conserved between mouse and human [45]. We have similarly observed that when using a biochemical approach (CLIP) [22], the majority of Nova-binding sites isolated from mouse brain are not conserved in the human genome ([22], unpublished observation). How can we reconcile the high conservation of the functional Nova binding sites analyzed in the current study with the much lower conservation of sites isolated via biochemical studies? We hypothesize that in addition to the highly conserved functional sites that were analyzed in this study, Nova might also bind less conserved sites, which could contribute to ability of the organism to evolve. To explore this question further in the future, biochemical analysis of Nova–RNA binding in brains of different species would need to be related to the alternative exons regulated by Nova in each species.

Taken together, the current work finds that brain-specific splicing patterns of Nova-regulated exons are highly conserved and are related to conservation of YCAY clusters in the pre-mRNAs. The presence of YCAY clusters is 100% predictive for the brain-specific alternative splicing of exons, whereas their absence significantly decreases the ratio of conserved splicing to 47%. The data also agree with the hypothesis that alternative splicing might in some cases evolve via addition of *cis*-acting sites that bind tissue-specific splicing factors such as Nova. Moreover, it is shown that the genes encoding adhesion and cytoskeletal proteins generally display deeper evolutionary fixation of YCAY clusters than the genes encoding receptors and signaling molecules, suggesting evolution of Nova function through evolution of target RNA sequences.

## Materials and Methods

### Genomic alignments.

We searched for human (Homo sapiens), opossum (Monodelphis domestica), chicken (Gallus gallus), frog (Xenopus tropicalis), and zebrafish (Danio rerio) genomic orthologous sequences with the Liftover tool on University of California Santa Cruz (UCSC) Genome Browser [46] using genomic data from March 2006 for human, February 2006 for mouse, January 2006 for opossum, May 2006 for chicken, August 2006 for frog, and March 2006 for zebrafish. We used the UCSC Table Browser [47] (http://genome.ucsc.edu/cgi-bin/hgTables) to download sequence flanking the splice sites; we analyzed 80 nucleotides of exonic sequence upstream of each 5′ splice site, 200 nucleotides of intronic sequence downstream of each 5′ splice site, 200 nucleotides of intronic sequence upstream of each 3′ splice site, and 80 nucleotides of exonic sequence downstream of each 3′ splice site. Sequences were further verified for proper alignment using MacVector ClustalW and T-Coffe multiple alignment tool, and cases where multiple possible alignments were present in the genome due to multiple paralogous matches were discarded.

### Calculation of YCAY cluster score.

We adjusted our original YCAY cluster score algorithm [24] for the purpose of this evolutionary study. Whereas the original paper [24] required stringent filtering of false positives due to analysis made on genomic scale, the current algorithm allowed for larger toleration in distance between YCAY motifs [22]. The majority of YCAY motifs are located in introns, which display fast mutation rates between such distant species as fish and mouse, and therefore the current algorithm was adapted to tolerate such mutations in a way that still detects the core feature of Nova binding site, i.e., three proximal YCAY motifs. In addition, the definition of the boundaries between the areas where the clusters act as silencers or enhancers was the same as in the previous study [24]. The YCAY cluster score was calculated by searching for the first YCAY motif, and then giving it a score relative to the pattern of YCAY motifs that followed it:

if YCAY[N>23]YCAY then s = 0

if YCAY[19<N<24]YCAY[[N<4]Y]CAY then s = 2

if YCAY[9<N<20]YCAY[3<N<20]YCAY then s = 2

if YCAY[9<N<20]YCAY[[N<4]Y]CAY then s = 4

if YCAY[3<N<10]YCAY[9<N<20]YCAY then s = 2

if YCAY[3<N<10]YCAY[3<N<10]YCAY then s = 4

if YCAY[3<N<10]YCAY[[N<4]Y]CAY then s = 6

if YCAY[[N<4]Y]CAY[19<N<24]YCAY then s = 2

if YCAY[[N<4]Y]CAY[9<N<20]YCAY then s = 4

if YCAY[[N<4]Y]CAY[3<N<10]YCAY then s = 6

if YCAY[[N<4]Y]CAY[[N<4]Y]CAY then s = 8

After the score is given to the first YCAY, the analysis moves on to the next YCAY, and so on in an iterative way until the end of the 45-nucleotide sequence window is reached. At that point, the score is calculated:

*S* = log(*s*1 + *s*2 + … + *sn*), where *n* is the number of YCAY motifs in the 45-nucleotide sequence window.

In order to predict the direction of Nova-dependent splicing regulation based on YCAY cluster position, net YCAY cluster score was calculated as described previously [24] using the following formula:

Net conserved S = 1/2(MAX(NISE1, NISE2, NISE3, SUM(NISE2, NISE3)*2/3) − MAX(NISS1, NISS2, NESE))

where NISE1 is nova intronic splicing enhancer 1, NISE2 is nova intronic splicing enhancer 2, NISE3 is nova intronic splicing enhancer 3, NISS1 is nova intronic splicing silencer 1, NISS2 is nova intronic splicing silencer 2, NESE is nova exonic splicing enhancer.

### RT-PCR.

Purified RNA from brain or liver of chicken (G. gallus) and zebrafish (D. rerio) was reverse transcribed using random hexamers, and cDNA products were amplified using *Taq* PCR Master Mix Kit (Qiagen) with 40 pmol of each primer and 0.5 pmol of one γ-^32^P-ATP-labeled primer at Tm = 55 °C. The primers used are listed in Table S6. PCR products were resolved on polyacrylamide gel electrophoresis and confirmed by size and sequencing.

### Western blot analysis.

Brain and liver from chicken (G. gallus) and zebrafish (D. rerio) were homogenized and protein concentration was determined with Bradford dye assay (BioRad). Proteins were separated by SDS/PAGE, transferred to nitrocellulose, and probed with rabbit polyclonal anti-Nova antibody that was made by immunization with full-length Nova1 [20] or rabbit polyclonal eIF3a (Santa Cruz). Blots were developed with horseradish peroxidase–linked secondary antibodies and enhanced chemiluminiescence (Amersham).

## Supporting Information

Figure S1 Alignment of Full-Length Nova1 and Nova2 Proteins in Four Different Vertebrate species Proteins were aligned using ClustalW (MacVector). KH domains are marked with red letters.
(366 KB PDF)

Figure S2 Alignments of YCAY Clusters and Gel Pictures of RT-PCR Results Raw data are shown for all the genes, for which pattern of alternative splicing was analyzed in chicken and/or zebrafish brain and liver mRNA. Alignments were made from orthologous sequences from eight species: human (H. sapiens), dog (Canis familiaris), mouse (Mus musculus), rat (Rattus norvegicus), opossum (M. domestica), chicken (G. gallus), frog (X. tropicalis), and zebrafish (D. rerio). Exon are capitalized and introns in small letters. The letters marked in light blue indicate intron/exon borders, YCAY sequences are marked with red, and the main mouse Nova binding element is indicated in grey. Below the alignment are results from RT-PCR experiments for chicken (G. gallus) and zebrafish (D. rerio), respectively. The picture also shows the structures of different mRNA isoforms with Nova binding site indicated (red circle for splicing enhancers and blue circle for splicing silencers).
Agrin: The main mouse Nova binding cluster is not present in chicken and zebrafish. But the splicing pattern of alternative exon, orthologous to mouse alternative exon 31a, in these two species is similar to that in mouse, where Nova regulated alternative exon 31a is more included in the brain. In both species, the alternative forms are weakly expressed.
Ank3: Mouse niss2 cluster is very well conserved in chicken, but it is not conserved in zebrafish. Splicing of chicken alternative exon, orthologous to mouse alternative exon 31, is conserved, with exon more excluded in brain (and more included in liver). We were not able to construct PCR primers for analyzing splicing of this alternative exon in zebrafish.
Aplp2: The main mouse YCAY cluster is conserved in chicken, but it is not present in zebrafish. Zebrafish has no detectable YCAY clusters in the downstream vicinity of the alternative exon. Splicing pattern of alternative exon, orthologous to mouse exon 12a, is conserved in both chicken and zebrafish, with alternative exon being more included in the brain. We also checked alternative splicing in opossum, which has a conserved YCAY cluster and also found conserved splicing pattern (unpublished data).
Brd9: The main mouse Nova binding cluster is extremely conserved in chicken and also very good in zebrafish. The splicing pattern of the chicken and zebrafish alternative exon, orthologous to mouse exon 5, is also conserved in both organisms, with alternative exon more included in the brain than in the liver. Note that the alternative exon 5 lies downstream of the YCAY cluster and is not shown in the alignment.
Cacna1b: The main mouse Nova binding cluster is conserved in chicken and zebrafish. The zebrafish cluster is located a little more upstream than in other organisms and so it is not visible in this alignment. Splicing pattern of the alternative exon, orthologous to exon 24a in mouse, is conserved in both chicken and zebrafish, with the exon more included in the brain.
Interestingly, it looks like zebrafish has a slightly different gene structure from other organisms, with an additional alternative exon adjacent to the known alternative exon.
Camk2g: The main mouse Nova binding cluster nise2 is not present in chicken and zebrafish (although it is conserved in human, dog, rat, and opossum). Chicken and zebrafish have a strong cluster located more downstream (approximately 100 nt further away from the alternative exon 13a than that of the mouse, which still corresponds to the nise2 area), which is not shown in this alignment. In chicken, the splicing pattern of the alternative exon, which is orthologous to mouse alternative exon 13a, is the same as in mouse, with the exon more included in the brain. In zebrafish, it looks as though the exon that is orthologous to mouse alternative exon 13b is more regulated, possibly because of the position of the cluster.
Clasp1: Mouse nese1 cluster is not conserved in chicken, but it is conserved in zebrafish (where the cluster is even stronger than in mouse, with five YCAY repeats). Interestingly, chicken has a splicing pattern more similar to that of the alternative exon, orthologous to mouse exon 9, than to that of the mouse, with the exon more included in the brain. We were not able to construct PCR primers for analyzing splicing of this alternative exon in zebrafish.
Clstn1: The main mouse YCAY cluster is not conserved in chicken or zebrafish. Interestingly, the chicken alternative exon, which is orthologous to mouse exon 10, has the same splicing pattern as in mouse, with the exon predominantly included in the brain. We were not able to construct PCR primers for analyzing splicing of this alternative exon in zebrafish.
Dab1: Mouse alternative exons 7b and 7c are preceded by niss2 elements. Exon 7b is not conserved in chicken, but it is conserved in zebrafish and the peptide sequence encoded by the two exons suggests that one of the two exons was generated via a duplication event during the evolution of mammals. Interestingly, these species also lack one of the niss2 elements, suggesting that duplication of the exon has also duplicated the upstream YCAY cluster. Splicing pattern of the conserved exon 7c in chicken is the same as in mouse, with the exon predominantly excluded in the brain. It looks as though in zebrafish both exons are conserved, with a conserved pattern of splicing (more often excluded in the brain). The gel has only three bands because both exons are probably the same length.
Efna5: The main mouse Nova binding cluster is conserved in chicken, but is not present in fish (where the whole area is actually missing). Splicing pattern of alternative exon, orthologous to mouse exon 3a, is conserved, with exon slightly more excluded in the brain than in the liver. Interestingly, the zebrafish alternative exon also shows a brain-specific splicing pattern, although the pattern is reversed, with the exon more included in the brain.
Epb4.1: The main mouse Nova binding cluster is conserved. Chicken has a weak YCAY cluster (the distance between the first and second YCAY is 17 nt and 0 nt between the second and third cluster, respectively), and zebrafish has a stronger cluster with five YCAY tetranucleotides (5 nt between the first and second YCAY, −1 between the second and third, 4 nt between third and fourth, and 3 nt between fourth and fifth). Splicing pattern of exon, orthologous to mouse alternative exon 14, is conserved in chicken, with the exon more included in the brain. In zebrafish, it looks like the exon orthologous to mouse alternative exon 13 has a conserved tissue-specific splicing pattern.
Epb41L2: Mouse alternative exon 12a and 12b are controlled by two YCAY clusters, nise2 and nise3. Neither of these clusters is conserved in the chicken (we could not obtain orthologous sequence for zebrafish), but chicken has one YCAY cluster in a very similar position to that of the mouse nise3 cluster. Both alternative exons in chicken display a tissue-specific splicing pattern, with the exon orthologous to mouse exon 12a more included in the liver, while the exon orthologous to mouse exon 12b is more included in the brain.
Gphn: Mouse niss1 cluster is not conserved in chicken or zebrafish. On the other hand, mouse niss2 and ness2 clusters are completely conserved in chicken (we could not get the orthologous sequence for zebrafish). Splicing pattern of the chicken exon orthologous to mouse alternative exon 7a is the same as in mouse, with the exon predominantly excluded in the brain (and predominantly included in liver).
Golga4: The main mouse Nova binding cluster is not conserved in chicken (we could not obtain orthologous sequence for zebrafish) and the exon orthologous to the mouse alternative exon is not present in chicken, so there is no alternative splicing present.
Kcnma1: The main mouse Nova binding cluster is located in the 5′ constitutive exon; in chicken, this YCAY cluster is not conserved, but in zebrafish, this cluster is very similar to that in mouse, with four YCAY motives (−1 nt between the first and second YCAY, 11 nt between the second and third, and −1 nt between the third and fourth). The splicing pattern of the alternative exon orthologous to mouse exon 24a is conserved both in chicken (even though here the YCAY cluster is not conserved) and zebrafish, with the exon more included in the brain.
Lrrfip1: The mouse nise1 element, located in alternative exon 17a, is conserved in chicken, but it is not present in zebrafish. Note that mouse alternative exon 17 acts as an alternative 3′ mRNA end. Splicing of the alternative exon orthologous to mouse exon 17a is conserved in chicken (even though the YCAY cluster is not conserved), with the exon more included in the brain. We were not able to construct PCR primers for analyzing splicing of this alternative exon in zebrafish.
Map4: The main mouse Nova binding cluster is not present in chicken and zebrafish. Chicken has similar splicing pattern to mouse (exon more excluded in brain, compared to liver), while in zebrafish there is only one form of the transcript (with exon included) and there is no splicing, even though the exon is conserved.
Map4k4: In mouse, Nova binds to two YCAY clusters in the vicinity of alternative exon 22a, niss1 and ness2. We obtained orthologous sequences only for the ness2 element, which is present in the alternative exon. The element is completely conserved in chicken, but is absent in zebrafish. In chicken, splicing of the exon orthologous to mouse alternative exon 22a follows the conservation of the ness2 element, with more exon exclusion in brain than in liver. We were not able to construct PCR primers for analyzing splicing of this alternative exon in zebrafish.
Neo1: The main mouse Nova binding cluster is located in alternative exon 27 and is almost perfectly conserved in all species (chicken and zebrafish actually have one additional YCAY sequence). The exon orthologous to mouse alternative exon 27 is predominantly excluded in the brain of chicken and zebrafish (slightly more in zebrafish [76% versus 90%]).
Plcl3: The main mouse Nova binding cluster is conserved in chicken, but is not conserved in zebrafish. Zebrafish has one YCAY cluster located very close (3 nt) to the 5′ end of the alternative exon (chicken: 4 nt between first and second YCAY, 1 nt between second and third YCAY; fish: 4 nt between first and second YCAY, 12 nt between second and third YCAY). Splicing of the exon orthologous to mouse alternative exon 22a is conserved in both chicken and zebrafish, with the exon more included in the brain.
Ptprf: The main mouse Nova binding cluster is present in chicken and zebrafish, with five YCAY tetranucleotides in zebrafish versus three in chicken. The splicing pattern in chicken and zebrafish of the exon orthologous to mouse alternative exon 6a is very similar to that in the mouse, where the exon is predominantly excluded outside brain.
Ptprf: Alternative exon 19a in mouse is regulated by two Nova binding sites, nise3 and nese2 element. Neither of the two elements is conserved in chicken or zebrafish (even though the element nese2 is located in the 5′ constitutive exon, which is strongly conserved). But there is also a conserved YCAY cluster, preceding the nise3 element, which is present in chicken but not in zebrafish. (We could not obtain the orthologous sequence from opossum, so we excluded this sequence from our nucleotide alignment). The splicing pattern of the chicken exon orthologous to mouse alternative exon 19a is the same as in mouse, where the exon is more included in the brain. We were not able to construct PCR primers for analyzing splicing of this alternative exon in zebrafish.
St7: Genomes of chicken and zebrafish does not have sequence orthologous to the mouse Nova-regulated alternative exon 7 and downstream sequence containing the Nova binding site. The PCR of chicken and zebrafish brain and liver showed only one form of this gene and the exon was not present.
Stxbp2: The main mouse Nova binding cluster is located on the border of alternative exon 3. The cluster is not conserved in either chicken or zebrafish. On the other hand, the exon orthologous to mouse alternative exon 3 is conserved in both organisms. The exon shows no alternative splicing in these two organisms and the only detected form is the one with the exon included.
Tacc2: The mouse nise3 cluster is not conserved in chicken (we could not obtain orthologous sequence for zebrafish).Although mouse alternative exon 10 is strongly conserved in chicken (even in exon length), it is not alternatively spliced but is constitutively included into mRNA. In addition, expression of the gene is low in liver. We were not able to construct PCR primers for analyzing splicing of this alternative exon in zebrafish.
(1.4 MB PDF)

Table S1 YCAY Cluster Scores of Orthologous Sequences within Pre-mRNAs That Were Identified by Splicing Microarray As Regulated by Nova in Mouse Brain Analysis of YCAY cluster scores in human (H. sapiens), opossum (M. domestica), chicken (G. gallus), frog (X. tropicalis), and zebrafish (D. rerio) sequences. In Table S1A, YCAY cluster score was calculated using the original algorithm parameters [24]. In Table S1B, YCAY cluster score was calculated according to the algorithm given in this paper. The YCAY cluster was considered conserved if the absolute value of the calculated cluster score was ≥0.6. For some mouse sequences we could not obtain orthologous opossum, chicken, frog, and zebrafish sequences. On the other hand, some mouse sequences (such as Golga4 and St7 sequences containing YCAY clusters) were not present in the genomes of other species.
(251 KB DOC)

Table S2 Scores of YCAY Clusters in Pre-mRNA of All Validated Mouse Nova-Regulated Exons Included in This Study, Together with Exon Inclusion Levels Seventy-six of YCAY clusters were assigned an absolute cluster score value ≥0.6 by analysis of mouse genomic sequence using our YCAY cluster score algorithm (see Materials and Methods).
(146 KB DOC)

Table S3 Expression Levels of Nova-Regulated Genes in Mouse Brain and Liver Expression levels (the median value of microarray signal in RNA from the tissue) of Nova-regulated genes were obtained from the Novartis GNF1m gene atlas (http://symatlas.gnf.org/) and in cases where there were no data for a particular gene, we searched for data from the splicing microarray of the Nova knockout mouse brain (http://splicing.rockefeller.edu/) and from the ArrayExpress repository for microarray data at the European Bioinformatics Institute (http://www.ebi.ac.uk/arrayexpress/). We assumed that genes with expression values <200 are not expressed in that tissue. We found 40 genes that had an expression value in liver >200. For the remaining genes that were interesting for our study but had an expression value <200, we manually checked their expression pattern in brain and liver with RT-PCR. From those genes, we could detect eight in the liver. For three genes (Brd9, Cp110, and Skip) there were no data about their expression in the above-mentioned databases. We examined their expression pattern with RT-PCR and found that Brd9 and Cp110 are expressed in brain and liver, while we could not detect Skip in the liver. Overall, we determined that from our list, 54 YCAY clusters were present in genes that are expressed in liver.
(197 KB DOC)

Table S4 Exon Length Conservation of 88 Mouse Nova Alternative Exons in Different Vertebrate Species The table shows exon length (in nucleotides) and predicted major/minor form (based on mRNA and EST data in mouse) of alternative exons validated as being regulated by Nova in mouse brain. Genomic conservation of alternative exons was determined with Liftover and Blat tools on the UCSC Genome Browser and in some cases confirmed with RT-PCR and sequencing. For some exons we could not determine the exon length (e.g., because there were multiple mRNA entries in the database with different lengths of the exon) or if they are conserved or not (e.g., genomic alignment indicated that the exon is conserved but then we could not reliably confirm that with Blat tool).
(227 KB DOC)

Table S5 Homology of Orthologous Alternative Exons The table shows percent identity between the mouse exon and the orthologous exon in different vertebrate species for all cases where we could obtain orthologous sequences.
(194 KB DOC)

Table S6 List of PCR Primer Sequences (75 KB DOC)

### Accession Numbers

The National Center for Biotechnology Information (NCBI) Entrez Gene (http://www.ncbi.nlm.nih.gov) accession numbers of the mouse genes discussed in this paper are: Neo, 4756; Stxbp2/Munc18–2, 81804; Ptprf, 5792; Aplp2, 11804; St7, 64213; Nova1, 18134; Nova2, 384569; GlyRα2, 237213; GluR6, 54257; neurochondrin, 26562; Lrp12, 239393; Gpr45, 93690; NR1, 14810; Napor, 14007; hnRNP A1, 15382; hnRNP H, 59013; and CaM kinase IV, 12326.

## Abbreviations

CLIP: cross-linking and immunoprecipitation
eIF3a: eukaryotic translation initiation factor 3A
EST: expressed sequence tag
KH: K-homology
Nova: neuro-oncological ventral antigen
