# Supplementary material for: Evolution of Nova-Dependent Splicing Regulation in the Brain
Source: PLoS Genet. 2007 Oct 12;3(10):e173. doi: 10.1371/journal.pgen.0030173 (PMC2014790; doi:10.1371/journal.pgen.0030173)
Supplement: Supplementary file 1 [file pgen.0030173.sg001.pdf]

A

Protein alignment of Nova1

```
                                                                                         K H 1    d o m a i n
human        MMAAAPIQQNGT-----HT--GVPIDLDPPDSRKRPLEAPPEAGSTKRTNTGEDGQYFLKVLIPSYAAGSIIGKGGQTIVQLQKETGATIKLSKSKDFYPGTTERVCLIQGTVEALNAVH
mouse        MMAAAPIHQNGT-----HT--GVPIDLDPPDSRKRPLEAPPEAGSTKRTNTGEDGQYFLKVLIPSYAAGSIIGKGGQTIVQLQKETGATIKLSKSKDFYPGTTERVCLIQGTIEALNAVH
chicken      MMAAAPIQQNGT-----HT--GVPIDLDPPDSRKRPLEAPPEAGSTKRTNTGEDGQYFLKVLIPSYAAGSIIGKGGQTIVQLQKETGATIKLSKSKDFYPGTTERVCLIQGTVEALNAVH
zebrafish    MMAGGEVQQNGIFSTPQHLSQSPQMDPDLSDSRKRPLETPTEASSTKRSNTGEEGEYFLKVLIPSYAAGSIIGKGGQTIVQLQKETGATIKLSKSKDFYPGTTERVCLIQGTVEALNGVH
Cons         ***.. ::***      *   .  :* * .********:*.**.****:****:*:********************************************************:****.**

                                                             K H 2      d o m a i n
human        GFIAEKIREMPQNVAKTEPVSILQPQTTVNPDRIKQVKIIVPNSTAGLIIGKGGATVKAVMEQSGAWVQLSQKPDGINLQERVVTVSGEPEQNRKAVELIIQKIQEDPQSGSCLNISYAN
mouse        GFIAEKIREMPQNVAKTEPVSILQPQTTVNPDRIKQVKIIVPNSTAGLIIGKGGATVKAIMEQSGAWVQLSQKPDGINLQERVVTVSGEPEQNRKAVELIIQKIQEDPQSGSCLNISYAN
chicken      GFIAEKIREMPQNVAKTEPVSILQPQTTVNPDRIKQVKIIVPNSTAGLIIGKGGATVKAIMEQSGAWVQLSQKPDGINLQERVVTVSGEPEQNRKAVELIIQKIQEDPQSGSCLNISYAN
zebrafish    NFIAEKVREMPQSSQKTEPVSILQPQTTVNPDRIKQAKLIVPNSTAGLIIGKGGATVKAVMEQSGAWVQLSQKPEGINLQERVVTVSGEPEQNRKAVEIIVQKIQEDPQSSSCLNISYSN
Cons         .*****:*****.  *********************.*:*******************:***************:***********************:*:*********.*******:*

human        VTGPVANSNPTGSPYANTAEVLPTAAAAAG---LLGHANLAGVAAFPAVLSGFTGNDLVAITSALNTLASYGYNLNTLGLGLSQAAATGALAAAAAASANPAAAAA-NLLATYASEASASG
mouse        VTGPVANSNPTGSPYANTAEVLPTAAAAAG---LLGHANLAGVAAFPAVLSGFTGNDLVAITSALNTLASYGYNLNTLGLGLSQAAATGALAAAAAASANPAAAAA-NLLATYASEASASG
chicken      VTGPVANSNPTGSPYANTAEVLPTAAAAAG---LLGHANLAGVAAFPAVLSGFTGNDLVAITSALNTLASYGYNLNTLGLGLSQAAATGALAAAAAASANPAAAAA-NLLATYASEASASG
zebrafish    ITGPVANSNPTGSPFANSTEVLPNAAAAATASTLLGQAGLAGMGGFPAAMSSLSGNDLLAITSALNTLASYGYNTNTLGLGLNPAAASGVLAAVAASANPAAAAAANLLATYASEASGGG
Cons         :*************:**::****.*****    ***:*.***:..***.:*.::****:*************** *******. ***:*.***.*********** ***********..*

                                                                            K H 3      d o m a i n
human        S-TAGGTAGTFALGSLAAATAATNGYFGAASPLAASAILGTEKSTDGSKDVVEIAVPENLVGAILGKGGKTLVEYQELTGARIQISKKGEFVPGTRNRKVTITGTPAATQAAQYLITQRI
mouse        S-TAGGTAGTFALGSLAAATAATNGYFGAASPLAASAILGTEKSTDGSKDVVEIAVPENLVGAILGKGGKTLVEYQELTGARIQISKKGEFVPGTRNRKVTITGTPAATQAAQYLITQRI
chicken      S-TAGGTAGTFALGSLAAATAATNGYFGAASPLAASAILGTEKSTDGSKDVVEIAVPENLVGAILGKGGKTLVEYQELTGARIQISKKGEFVPGTRNRKVTITGTPAATQAAQYLITQRI
zebrafish    GHPAAPSLGGFSLGSLAAATGASNGYLNPSSPLVASSLLGTEKLAEGGKEVVEIAVPENLVGAILGKGGKTLVEYQELTGARIQISKKGEFIPGTRNRKVTITGSPAATQAAQYLISQRI
Cons         . .*. : * *:********.*:***:..:***.**::***** ::*.*:*****************************************:************:***********:***

human        TYEQGVRAANPQKVG
mouse        TYEQGVRAANPQKVG
chicken      TYEQGVRAANPQKVG
zebrafish    TYEQGVRATNPQKVG
Cons         ********:******
```

B

Protein alignment of Nova2

```
human      ------------------------MEPEAPDSRKRPLETPPEVVCTKRSNTG------------------------------------------------------------------------
mouse      -----MGAQKGVALTA--------MGPEAPDSRKRPLETPPEVVCTKRSNTGGEAKLRDRGVGRGGGSRAEGGIAVLHHPPPSLAGRCKCFREGRGTPPRGDGDPDGPLSLVAEAPGAES
chicken    MMAAAPIQQNGT------HTGVP-IDLDPPDSRKRPLEAPPEAGSTKRTNTG------------------------------------------------------------------------
zebrafish  -MAGGAVQQNGIFSNPHHHNQQPHMESDPPDSRKRPLETPTEASSTKRTNTGDRCNQ-----------------AAIHDPP---------------------------------------
Cons                               :  :.*********:*.*.  .***:***

                                                KH1 domain
human      ------------EEGEYFLKVLIPSYAAGSIIGKGGQTIVQLQKETGATIKLSKSKDFYPGTTERVCLVQGTAEALNAVHSFIAEKVREIPQAMTKPEVVNILQPQTTMNPDRAKQAKLI
mouse      FVCDERGWGGGGEEGEYFLKVLIPSYAAGSIIGKGGQTIVQLQKETGATIKLSKSKDFYPGTTERVCLVQGTAEALNAVHSFIAEKVREIPQAMTKPEVVNILQPQTTMNPDRAKQAKLI
chicken    ------------EDGQYFLKVLIPSYAAGSIIGKGGQTIVQLQKETGATIKLSKSKDFYPGTTERVCLIQGTVEALNAVHGFIAEKIREMPQNVAKTEPVSILQPQTTVNPDRIKQVKII
zebrafish  FSSDIVGF---HEEGEYFLKVLIPSYAAGSIIGKGGQTIVQLQKETGATIKLSKSKDFYPGTTERVCLIQGTVEALNNVHDFIAEKVREMPQSAQKTEPVSILQPQTTVNPDRVKQAKLI
Cons                   *:*:*****************************************************:***.**** **.*****:**:**   *.* *.*******:**** **.*:*

                                 KH2 domain
human      VPNSTAGLIIGKGGATVKAVMEQSGAWVQLSQKPEGINLQERVVTVSGEPEQVHKAVSAIVQKVQEDPQSSSCLNISYANVAGPVANSNPTGSPYASPADVLPAAAAASAAAASGLLGPA
mouse      VPNSTAGLIIGKGGATVKAVMEQSGAWVQLSQKPEGINLQERVVTVSGEPEQVHKAVSAIVQKVQEDPQSSSCLNISYANVAGPVANSNPTGSPYASPADVLPAAAAASAAAASGLLGPA
chicken    VPNSTAGLIIGKGGATVKAIMEQSGAWVQLSQKPDGINLQERVVTVSGEPEQNRKAVELIIQKIQEDPQSGSCLNISYANVTGPVANSNPTGSPYANTAEVLP-----TAAAAAGLLGHA
zebrafish  VPNSTAGLIIGKGGATVKAVMEQSGAWVQLSQKPDGINLQERVVTISGEPEQNRKAVEIIVQKIQEDPQSSSCLNISYSNISGPVANSNPTGSPYANSAEVMPAAAAAAAATASSLLGQA
Cons       ******************:**************:**********:******  :***.  *:**:******.*******:*::**************..*:*:*     :**:*:.*** *

human      GLAGVGAFPAALPAFSGTDLLAISTALNTLASYGYNTNSLGLGLNSAAASGVLAAVAAGANPAAAAAANLLASYAGEAGAGPAGGAAPPPPPPPGALGSFALA------AAANGYLGAGA
mouse      GLAGVGAFPAALPAFSGTDLLAISTALNTLASYGYNTNSLSLGLNSAAASGVLAAVAAGANPAAAAAANLLASYAGDAGAGPGAGAAPPPPPPPGALGSFALAAA------ANGYLGAGA
chicken    NLAGVAAFPAVLSGFTGNDLVAITSALNTLASYGYNLNTLGLGLSQAAATGALAAAAASANPAAA-AANLLATYASEASAS---GSTAGGTAGTFALGSLAAA-----TAATNGYFGAA-
zebrafish  SLAGVGAFPTTMSSFSGNDLLAITSALNTLASYGYNTNSLGLGLNPAAASGVLAAVAASANPAAAAAANLLASYANDASTSAG--------HPATSLGGFTLGSLAAATGATNGYLSAAS
Cons       .****.***:.:..*:*.**:**::***********  *:*.***.  ***:*.***.**.****** ******:**.:*.:.            .  :**.:: .         :***:.*.

                                                                                 KH3 domain
human      GGGAGGGGGPLVAAAAAAGAAGGFLTAEKLAAESAKELVEIAVPENLVGAILGKGGKTLVEYQELTGARIQISKKGEFLPGTRNRRVTITGSPAATQAAQYLISQRVTYEQGVRASNPQK
mouse      GGAAGAGGAPLVAAAAAAGAAGGFLTAEKLAAESAKELVEIAVPENLVGAILGKGGKTLVEYQELTGARIQISKKGEFLPGTRNRRVTITGSPAATQAAQYLISQRVTYEQGVRASNPQK
chicken    --------SPLAASA--------ILG-TEKSTDGSKDVVEIAVPENLVGAILGKGGKTLVEYQELTGARIQISKKGEFVPGTRNRKVTITGTPAATQAAQYLITQRITYEQGVRAANPQK
zebrafish  -----------------PLVASSLLATE-KLAEGAKDVVEIAVPENLVGAILGKGGKTLVEYQELTGARIQISKKGEFVPGTRNRKVTITGSPAATQAAQYLISQRITYEQGVRATNPQK
Cons                               :*       ::.:*::****************************************:******:*****:***********:**:********:****
```
