# Supplementary material for: Evolution of Nova-Dependent Splicing Regulation in the Brain
Source: PLoS Genet. 2007 Oct 12;3(10):e173. doi: 10.1371/journal.pgen.0030173 (PMC2014790; doi:10.1371/journal.pgen.0030173)
Supplement: Supplementary file 2 [file pgen.0030173.sg002.pdf]

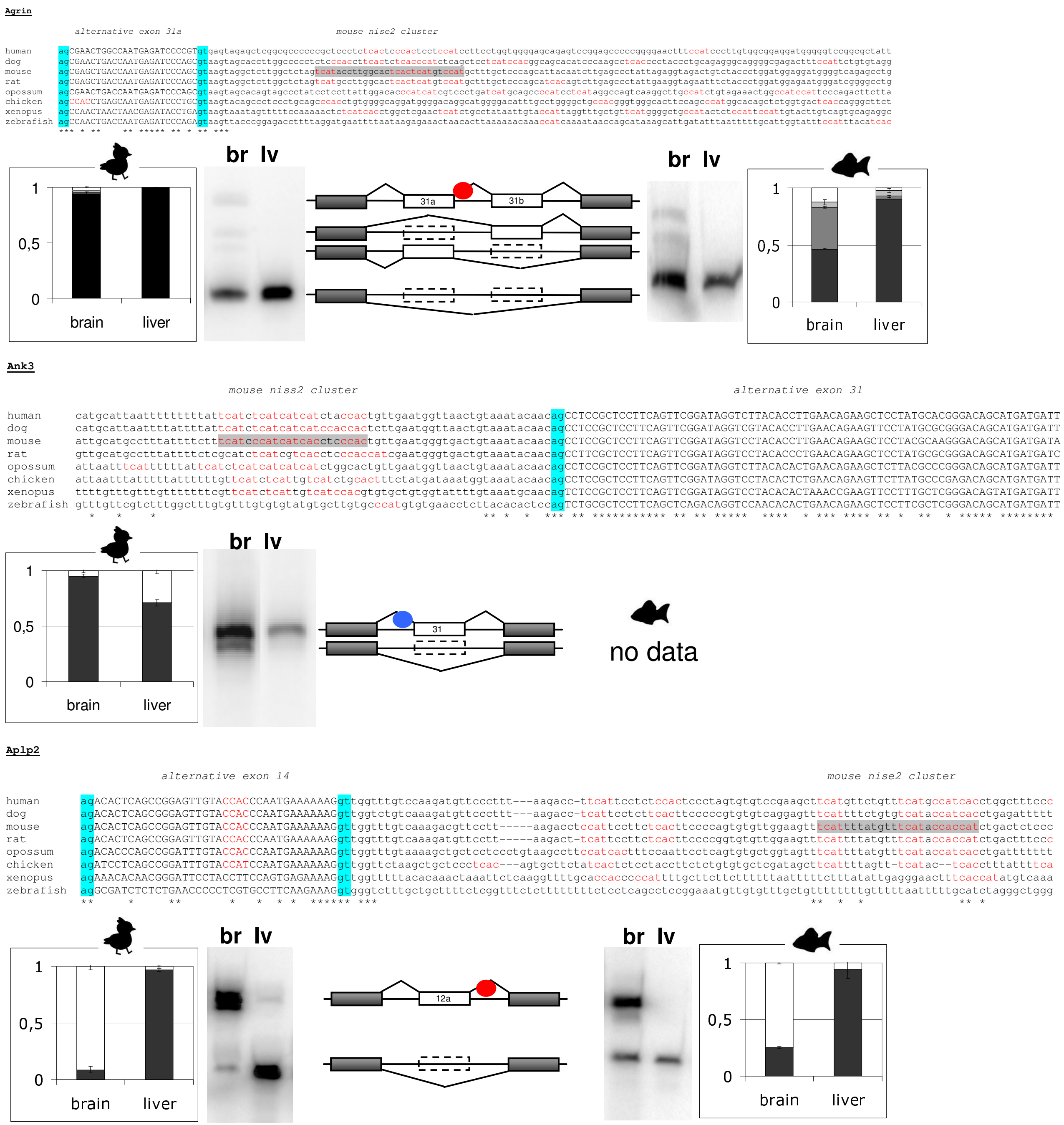
Agrin
alternative exon 31a
mouse nise2 cluster
human
dog
mouse
rat
opossum
chicken
xenopus
zebrafish
br lv
1
0,5
0
brain
liver
31a
31b
Ank3
mouse niss2 cluster
alternative exon 31
human
dog
mouse
rat
opossum
chicken
xenopus
zebrafish
br lv
1
0,5
0
brain
liver
31
no data
Aplp2
alternative exon 14
mouse nise2 cluster
human
dog
mouse
rat
opossum
chicken
xenopus
zebrafish
br lv
1
0,5
0
brain
liver
12a

Brd9

mouse nise1 cluster

```
human    gctcttagg--ccag--gtga-ggctgtgaccatccccatcccatcccatcgtcacgg-caccctg--g---ctgtgagggagaggtaaat-----ccctgctgctctgtcctt--tctg--attctaggagcgg
dog      gcggttagg--ccag--gtga-ggctg-ggccatccccatccca-cccatcgtcatgg-caccctg--g----tgtggggcagaggtaaag-----ccctgctgctctgtcctt--tttg--attctaggagcgg
mouse    gcgattagg--ccag--gtga-ggccgtggccat-cccatgccatcccatcgtcacgg-ctccgtg--g---c--tgagggagaggtaaag-----tcctgctgctctgtcctt--tctg--attctaggagcgg
rat      gcgattagg--ccag--gtga-ggccgtggccat-cccatgccatcccatcgtcacgggctccgtg--g---c--tgagggagaggtaaag-----tcctgctgctctgtcctt--tctg--attctaggagcgg
opossum  gccattaga--aatgaagtaagggccatggccattccccatcccatcccattctcatag-cgccttg--g---ctatgcgggaaaggtaaag----tccctgccgctctgtcctt--tctg--attctaggaacgg
chicken  ggcataaagaacaaa--ataa-ggccatggccattccccatcccatcccattatcatag-catcttg--g---ctatgagggaaaggttaag----tccctgccgctctgtcctt--tctg--attctaggaacgg
xenopus  c-----agc-ttatt--gtga-ggcat-tgccattccccatcacatccattcatcatag-cacctttt-tg---ctatgattgaaatgtaaag----tccctgctgctctgtcctt--ttttaattctaggaacgg
zebrafish------------------tctg-gttttcgccatttgcgcgtc-tcccatcttcatgg-cataccgaaggcctcccgcaggaggagtaaatgggtgctgtgttgctctatttgtgctctg--ttcacaggagcgg
                           *    *      ****   *    *  **  *  ***  * *        *       *    *   ** **         **   ***** *   *  * *    *  **** ***
```

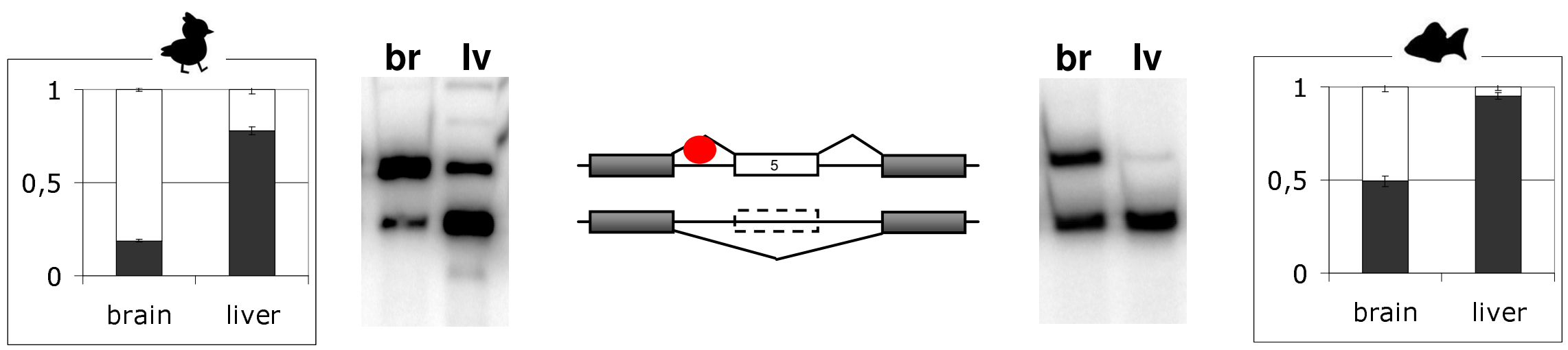

Cacna1b

alternative exon 24a *mouse nise2 cluster*

```
human     agGAGCTTCGTGGGgtaatgccttctctccccccacacgctgcccctgacatcacagcattccagcagcccctcttgggtcatttcatctccagcctcacccccgcccatccactttcaccccatcggggctgcatgctgccggg
dog       agGAGCTTCATGGGgtaatgccttccctctgccccttacactgccccgcatcatagcaaagcctggctccttctgggccagttcaccaccatgcctcacccttgctcatcctcctccactgccagggtgcatgctgccaggagcc
mouse     agGAGCTTCATGGGgtaatgcctcccctttctttcatattgccttacatcatggcacgcctctggcccatccatcatggcctcacacctgctcatctacctccaccctgtttaggctgcatgcttcctcccaggagccatcccca
rat       agGAGCTTCATGGGgtaatgtctcccttcccttgacattgccttacatcatggcacgcctctggcctttggcccatccatcatggcctcacacctgctcatccacctccaccctctttaggctgcatgctcccccaggagccat
opossum   agGAGCTTTATGGGgtaatgcgttcttgctgtctctcctgccctactaataatgcctgtgcactctcacacacccttagcaaaccacatcccaggcaacatggctgtaatccatgttatcctcattccgaatcaattggattcca
chicken   agGAGTTTCATGGGgtaatatgttattcctgtatcttgctgcactgttatgcctgtcaaatgtgtgtacacacacttaccactgtggaaatggttcatgttacacccattgcatcgatccctctgtcagttatcttaacgctgtc
xenopus   agGAGTTTTGTTGGgtaaattgaatctcctttctctgcacatacagcactgctctgtatgggagcacatgttttgttttttttcaaacaacccataatgtagacgcattgcaatgccttcatccgtcccccagaggtagagaaac
zebrafish agGAGCTTCATGGGgtaacgccttaactctctctgtctttgtttctgtttctgtctctttctttcactgttcgtttctttcctgtctctatgtgagaacacttagcttagctcatagacaagcattattcaagcatggattctt
          ***** **  * ******
```

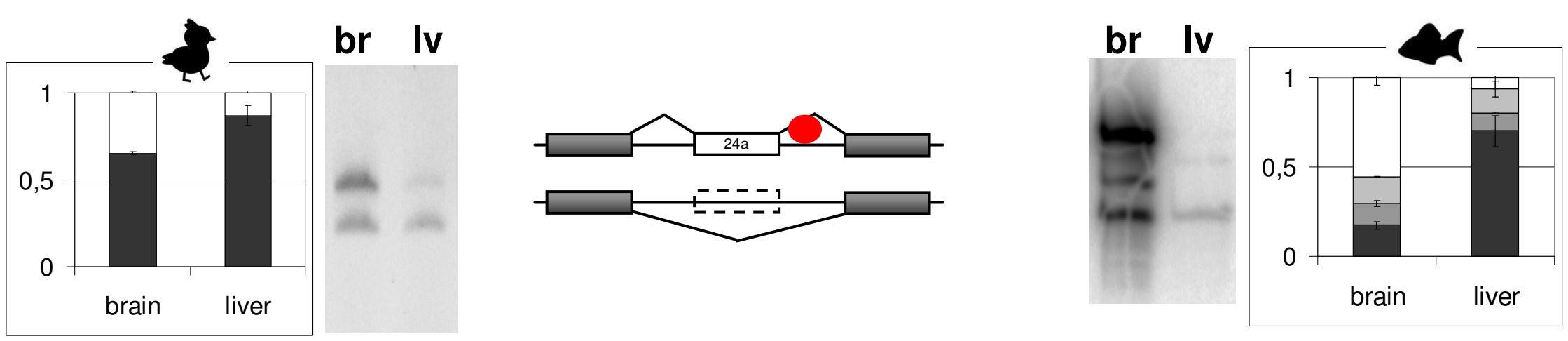

Camk2g

alternative exon 13a *mouse nise2 cluster*

```
human     agAAAAGGAAGTCGAGTTCCAGCGTGCACCTAATGgtgagccttgcctgcccacccatcgcccactccatgctgcctgtgcccgcctgccagccacgcaaacctgttctgccacgtgcgtgtgcctcactcatcctcactgcatg
dog       agAAAAGGAAGTCGAGTTCCAGCGTGCACCTAATGgtgagccttgcctgcccacccacctgcctatccatgctgcctgcgcccgcctgccagccacgcaaacccattctgccatgtgcgtgtgcttcactcatcctcactgcatg
mouse     agAAAAGGAAGTCGAGTTCCAGCGTGCACCTAATGgtgagccttgcctgcccacccaccctccacttcatgctgcttgtgcccgcctgccagccatgcagacccattctgccatgtgcgtgtgcgtcactcatcctcactgcatg
rat       agAAAAGGAAGTCGAGTTCCAGCGTGCACCTAATGgtgagccttgcctgcccacccaccccccactccatgctgcttgtgcccgcctgccagccatgcagacccattctgccatgtgcgcgtgcgtccctcatcctcactgcatg
opossum   agAAAAGGAAGTCGAGTTCCAGCGTGCACCTGATGgtgagcccggcctgcccacccatctgcccaccccctgctgcccgcccgcccgccatgcatattcatcctcatgccatgcacgtctgcttgtgctcattaccaccacatct
chicken   agAAAAGAAAGTCCAGTTCCAGCGTTCAGTTAATGgtgagtgtccttcgtcttcctcaatgccgatattgctgggcctttccacacgttccagccttccccgttttggaagttacctcggggtagggaagaagggccaccggg
zebrafish agAAAAGGAAGTCAAGCTCCAGTGTTTATTTGATGgtgagatctgttccgtgtctctccccttttcctgactctccctcaacactttctcgctctctctctctttgtttttttttttttcttggttgagatgctcgagagttt
          ******* ***** ** ***** **  *  * ********                                         *                   *    *
```

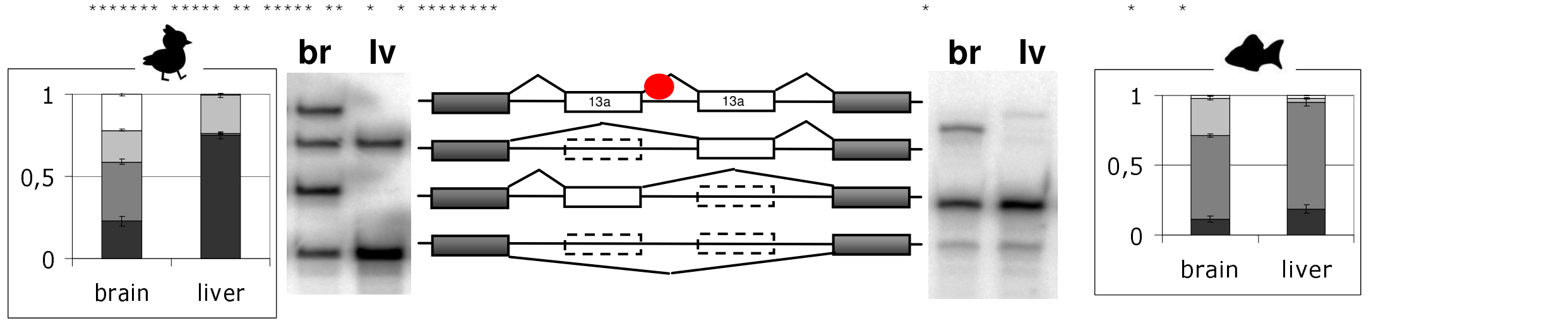

Clasp1

```
                                    5' constitutive exon                             mouse nese1 cluster

human      agGGCTCTCCAAGCAATACGATTGGCCGGACGCCCTCCCGACACACCAGCAGCAGGACCAGCCCCCTGACCTCACCCACCAACTGTTCCCATGGGGGTCTGTCTCCAAGgtaaggtcacttctttactttccacagatgtaaaga
dog        agGGCTCTCCGAGCAATACAATTGGCCGGACTCCCTCCCGACACACCAGTAGCAGGACCAGCCCCTTGACCTCACCCACCAACTGTTCCCATGGGGGCCTATCTCCCAGgtaatgccatttcaagaaaagtgaagaaactaaagc
mouse      agGGATCTCCAAGCAATACAATTGGCCGGACACCTTCCCGCCACCCCAGCAGCAGGACCAGCCCCCTGACCTCACCCACCAACTGTTCCCATGGGGGACTATCTCCAAGgtaagctcattccacagaagtgaaaaatgaaagtgc
rat        agGGATCTCCAAGCAATACCATTGGCCGGACACCTTCCCGACACCCCAGTAGCAGGACCAGCCCCTTGACCTCACCCACCAACTGTTCCCATGGGGGACTATCTCCAAGgtaagctcattccacaggagtgaaaaatgaaagtgc
opossum    agGGCTCTCCAAGCAATACCATTGGTCGAACTCCTTCAAGACACACCAGTAGCAGGACTAGCCCCTTGACTTCACCTACCAACTGTTCCCATGGTGGTCTGTCACCAAGgtaacaataagatttttaactaaaatggtctaatac
chicken    agGGTTCCCCCAGCAATACTCTTGGTAGGACTCCCTCTCGGCACTCCAGCAGCAGAACCAGCCCCTTGACTTCACCTACCAACTGTTCCCATGGTGGGCTGTCTCCAAGgtaatgtggcatgttaattaaacttaaaaaaaaaa
xenopus    agGGATCCCCGAGCAACACCATTGGCCGAACTCCTTCCAGGCACTCGAGCAGCCGCGCCAGCCCCCTCACATCTCCCACCAATTGCTCCCACGGCGGCCTCTCCCCCAGgtaacacagtgcggcttcctgcctgcagccaacctg
zebrafish  agAGTTCACCAAGCAATACAATGGGCCGCACACCAGCCCGGCACCCCACCAGCAGGACTAGTCCACTCACCTCACCCACAAACTGCTCTCATGGAGGTCTTTCGCCCAGgtacacgcacattatcatccgttcctccacacttat
           ** * ** ** ***** **  * **  * ** **  *  * *** * *  *** *  * ** **  * ** ** ** ** ** ** ** ** ** ** ** ** ** ** *****
```

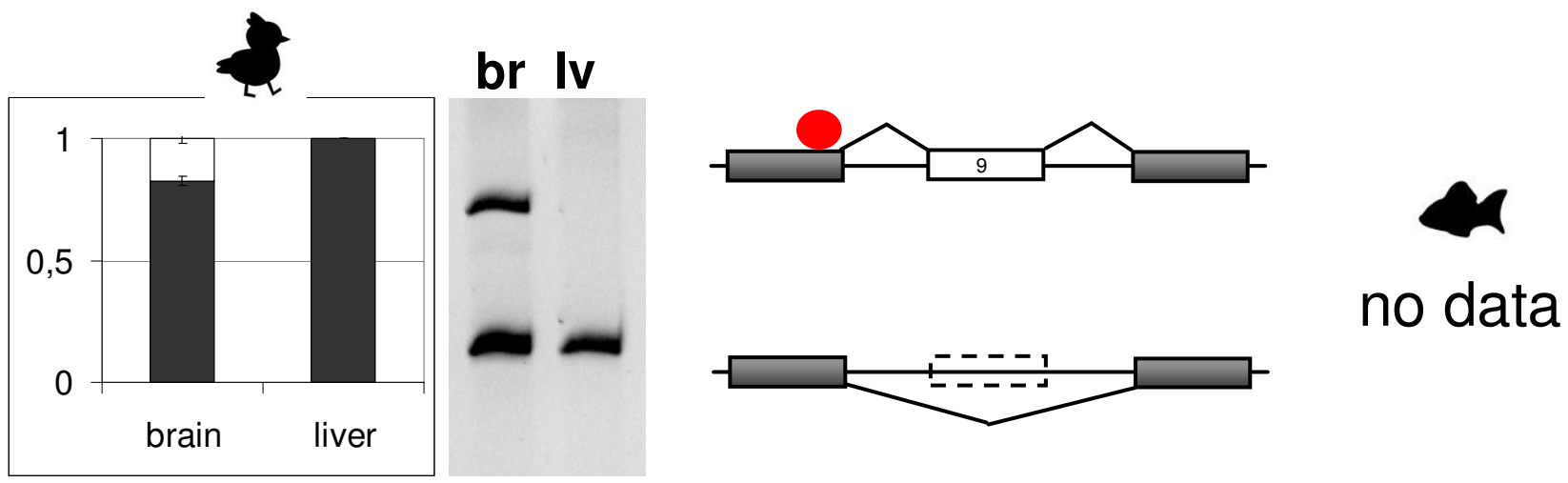

Clstn1

```
                                 alternative exon 10                                   mouse nise2 cluster

human      agAGTTTTCAGGAGTTGAAAATGACAATGAAACTGAGCCTG------TGACTGTGGCCTCTGCAGgttgctggatttttccctacattcactattgtgtttcatttaaagatgagactgattcattccacgtgtcgcatg
dog        agAGTATCCAGCATTTGAAAATGACAATGAAACCGAACCTG------TGCCTATGGCCTCTGCAGgttgctggatctttccctacggttcactcttgtgtttcatttaaagacgagatatcaattcattgcacatttcgca
mouse      agAGTATTCAGGAGTGGAGAGTGGCAATGAGACTGAGCCTG------CGACGATGGCCTCTGCAGgttgatggatcacccccacccccacccgcattccccattcactatagcgtttcctttaaagatgagacgttgatcca
rat        agAGTACTCAGGAGTCGAAAGTGGCAATGAGACTGAGCCTG------TGACGATGGCCTCTGCAGgttgctggatcacccccacccccacccgcactccccttcactatagtgcttcctttaaagatgagacattgatcca
opossum    agAGTATTCAGGAAATGAAAATGACAATGAGACTGAGGCTGAGGCAGTGACTGTGACCTCTGCAGgttgctggagttctctcctcctgttttctatcttatttccctttaagagacaagacaccaactcattccacatttg
chicken    agAATATACAGGAAATGAAAATGACAATGAAACCC------------TGCCTGAGACCTCTGCAGgttgctggagtttttccctactgttttatgtcattttaagccaagacattgcttcatctcacagcatgaaatgc
zebrafish  agACTTCTCAGGACATGACAATGACACTGAGACTCTCTCAG------AGCCTTTGA------CAGgttgccc-atgtctttcctgatctcaccaatctatccaaacgcacgtctcttaacgcatatgctcagcttcagtcg
           *** *   *** *   ** * ** ** *** **               * *   *       *******    *
```

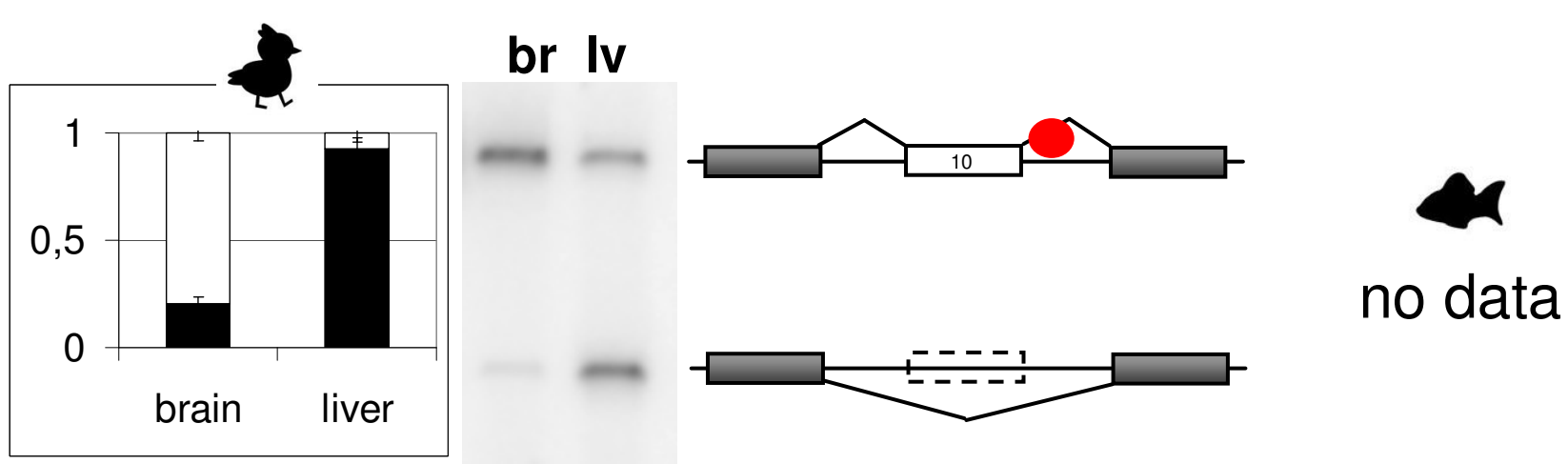

Dab1

```
                               mouse niss2 cluster                                            alternative exon 7c

human    -tggtta-actgtttcatgtcatttttctcatcattcatttcatgctcatcagtcatgccttgtggttacagAACAGAAACCTGCCCACAGACTTTGATGAGATTTTTGA-GG-C--AACGA---AGgt
dog      -tgatta-actgtttcaggtcatgatcccatcgttcatttcatgctcataagtcgtgccttgtggttacagAACAGAAACCTGCCGATGGACTTTGATGAGATTTTAGA-GG-C--AACAA---AGgt
mouse    -tgttta-actgtttcatgttgtcggaccatcactcattctgtgctcatccatcatgccttgtggttgcagAACAGGAACCTGTCAATGGACTTTGATGAGCTTCTCGA-GG-C--AACCA---AGgt
rat      -tgttta-actgtctcgtgttgtcggaccatcactcattctatgctcatccatcatgccttgtggttgcagAACGGAAACCTGTCAATGGACTTTGATGAGCTTCTTGA-GG-C--AACCA---AGgt
opossum  -caattc-atttattgcaattgtcttcatgtcattcatttcattttcatacatcatgccttgtggtttcagAACCGAAACTCAATGGTGGACTTTGACGAGTGT-TACAGGGTCGAAAATC---AGgt
chicken  atgctcaga-aattcatgtcatttttcataaatcattcatttcatttcataaatcatgctttacagctggagAATGGAAACTTATTGCTGGACATTGATGAAAATCTTG--GTTC--AGTCACTCAGgt
            *   *            *           *   *         ****   ** *** **   * *  ****  * ***          *** **** **    * *    *  *  *        ****
```

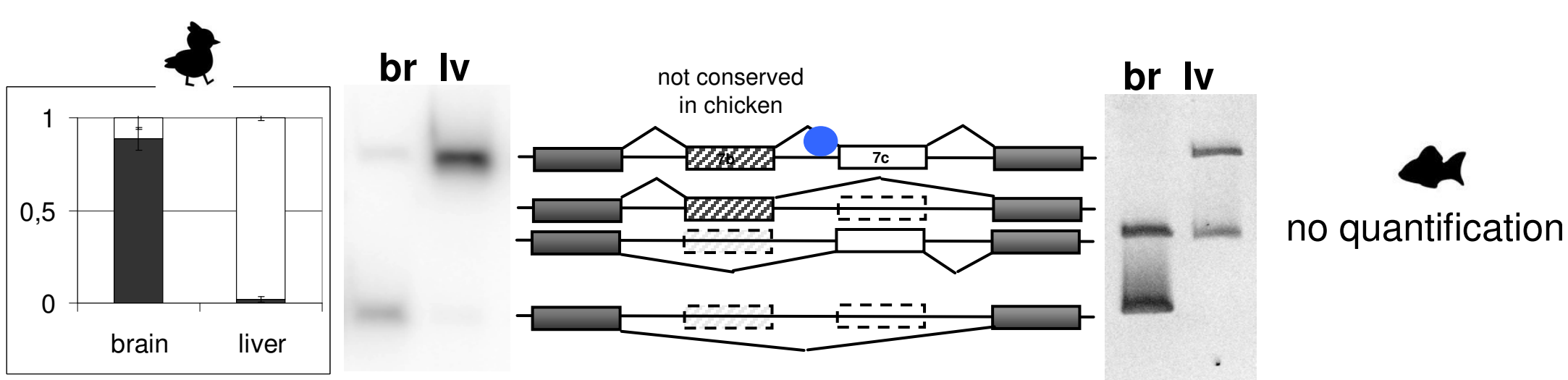

Efna5

mouse niss1 cluster

```
human      t-ttt----------------------tt-actgttaaatct--tgtgtggtatctgttttgtatatcaacattcatattttagttttc--atcatatttt-------ttctcattcttcatgatgcttttac-----------
dog        t-ttttttt----tttct---ttttttttttaactgttaaatcctgtgtgtggtatccgttttgtatatcggcatccgtggtttagttttc-accatgttttttttttccccatcatccttcgtgatgctttt--------attt-
mouse      tgtttcattctgttttatctatttattttt-actgtcgagacg--tgtgtggtacctgttttgtatatccacatccatgtttgagttttcc-atcatattttttttt----cctcattcctcatgatgcttttt------atttc
rat        tttattatt---atttct-tttttttttt-actgtcaagact--tgtgtgatccctgttatgtatatccacatccacatttgagtttcccatcgtatttcc-------cctcattcttcacgatgcttttttttttttatttc
opossum    t--------------------------tt-actgttaaaccc--tctgtggtatctgttctgtatatcagcatcatatttcagttttc--atcatatttttc-----ttatcattcttcataaagcttttta----------
chicken    -ttttt------ttttct------tttttt-atggttaagcct--tgtgt---atctgttctgtacagcagcatttgtgttactgttttc--atggtatttc------cttatcattttttcatgaagctttgtttt---------
xenopus    --------------ttct-----agttttt-actgttacgcct--tgtttgaatcttgttc------tcaacatttgtggtttagtttgc--accatatttc-------taatcattgttcattgagcttta-------------
zebrafish  ----------------------ctcacttta-atggacactaatggctctggctctcctttt---------------------------------------------------------------------------------------
                                 **     *                  * *         **
```

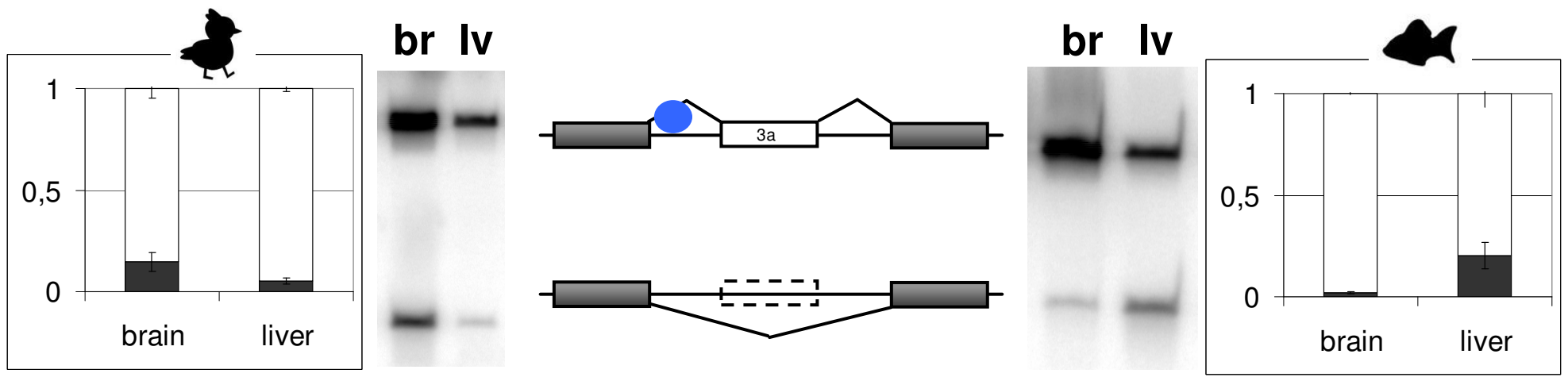

Epb4.1

alternative exon 14 *mouse nise2 cluster*

```
human      agAAGCT---TGCAGAAAAAACTGAAGATCTGATAAGAATGAGGAAGgttagccattttttc------actttcacaaaactacat---tgccatttcacccatgctgact--t-acag-tgcattaaaaaga---cagtgat---
dog        agAAGCT---TGCAGAAAAAACTGAAGATCTGATAAGAATGAGGAAGgtcagccattttttc------actttcacaaaaccacat---tac--tgtcacccatgctgacttct-acag-tgcattaaaaaga---cagtgat---
mouse      agAAGCT---TGCAGGAAAAGGTGAAGATCTGATAAGAATGAGGAAGgttagcctattttc------cctttcaca--gctaggc---caccattccacccatgc---ccgtt-acag-tgtattaaaaaga---cagtggt---
rat        agAAGCT---TGCAGAAAAAGGTGAAGATCTGATAAGAATGAGGAAGgttagcctgttttc------actttcaca--gctaggc---caccatttcacccatgc---ccgct-acag-tgtattaaaaaga---cagtggt---
chicken    agAAGCAGCCTGCAGAAAAAGAGGAAGAGCTGATAAGAATGAGGAAGgtcagccgttttac------gctttcaccgagcaaatt---tgccgtatcactcatgcatc---ct-cgacctgt-ttaaaaaga---cagtgct---
xenopus    agCAGCA---AGCTGAGAAAGAGGATGAAATAGTACGCATGAGGAAGgtcagcatagtgtc------tgttgaagtcaatctgtgtctttccatctctcacataaggaaagc-----aaaacatttacatcatttcat-----gt
zebrafish  ag------TCGCCACGGGTGGACACAGAGATGGTGCGCATGAGGAAGgtcagagcttgttccgtacgatttttcacagcatgaggaagatgccattgtactcatcatctc--ctcactg-tccatgaaaaaaa---aaaaagaaaa
           **     *              ** *  *  * *********** **        *         ** *                 *  *    * ***                    *  * *   *    *
```

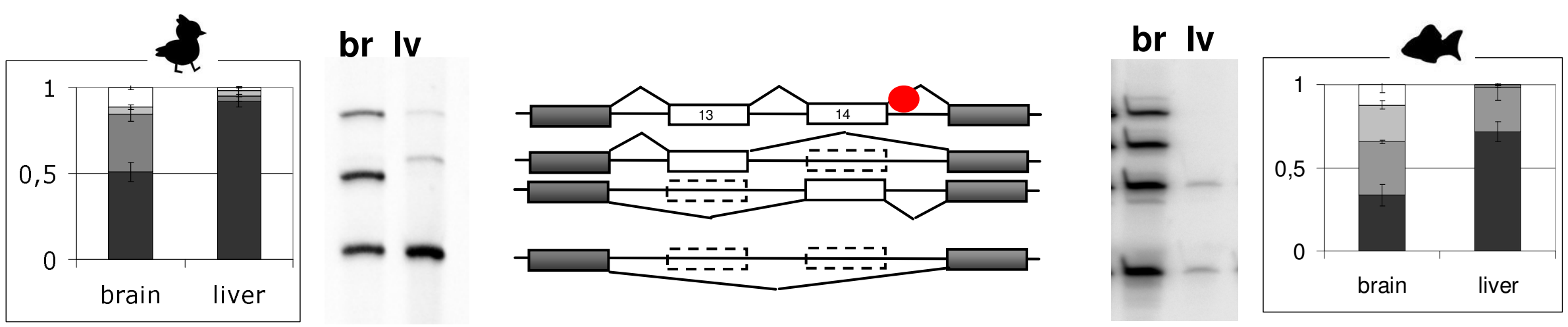

Epb41L2

mouse nise2 cluster

```
human      gttctcccc-tttttccat-gttaacaccattgc--ctcacgtttctcattgccctcacgtttgccc-cgcttccctttcagtcagc--c-a-----t
dog        gttctcccc-tt-ttccat-gttaacaccattgc--ctgatgtttctgactccccgct-----cccc-tgcttccctttcagtcagc--c-a-----t
mouse      gttctcctt------ccat-gttaatgccattcc--ctcacacctctcatccgcctcatggt---ca-cgcttccctttcagtccgca---------t
rat        gttctcctc------tcat-gctaatgccgttcc--ctcacgcctctcatc-ccctcgtgt-----c-cccttccctttcagtccgcatc-acagtgt
opossum    gttctccca-tttttccat-gttaattccattgc--ctgccatgtctgatcacctcacatttcccccctcttccctttcagtcagc--cca-----t
chicken    gttctactgatttttccatagttaaa---tttggctctaactcttccgatc-----catctt---ca-ttctttcttttcagttaac--c-a-----t
xenopus    gtgctcata-ttgttccata---agtactaatgtat-tcatcttaatcatc---accacattt--------ttttctttcacttttca-------gtt
           ** **          ***    *       *     *      *     *          **   ***** *   *          *
```

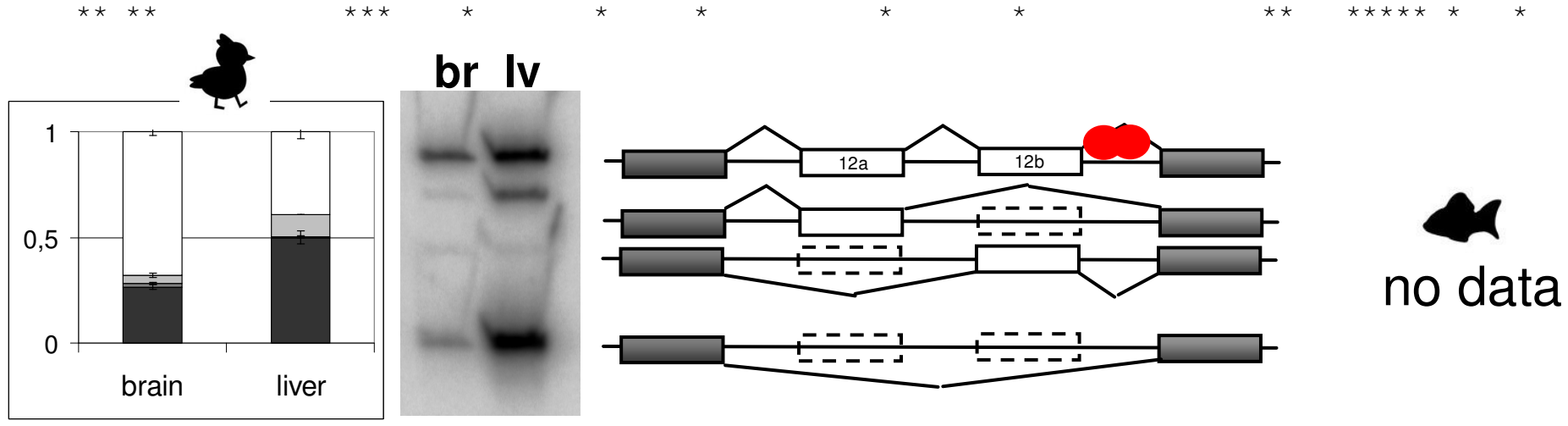

Golga4

```
                                                   mouse nise2 cluster

human   ttag------aatactaccacctgattttgtatgtaag--cactgaagttgtta-------tcctacttatttcat-aagtg----------------gtatcttct-ttcctctctgtctccct-caaaacatctcatcttga
dog     tcaggggagaagcactagcacctgattttgtatgtaag--cactgaacttgttaa------tcctacttatttcgt-gagtgtatcttatttcataaggtatcttct-ttcctctctgtctccct-caaaacacctcatcttga
mouse   ccaggagag----agcaccacctgacctgagatgtaag--cactgagctcatta-------ccattctcacctcat-gt-tg----------------acatcttct-ctcctctctgtctgcct-aaccacatctcatcttga
rat     ccaggagag----agtaccacctgacctgagatgtaag--cactggactcgtta-------tccttctcatttcat-aa-tg----------------acatcttct-ctcctctctgtcctcca-aacaacatttcatcttga
opossum ct----gac----agctggacatgattttatatttaaa--cactgaatttgtta-------tc-ttct--tttcat-aagtg----------------gtgtcttct-ttcctctctgtttccct-caaaactcctcaccttga
chicken acatgtgag-----gctggtaataa----acagttgacttca-ag-agcagcaatcttatcgcatccaca-ttcttgaagag--------------------caacagctact-gct-tctgtagcaatcacat-tctgtttga
                 * *      *  * *    **  *         *         * * *     ** *     *                    *   *    *  **   ** *         *  **    **    ****
```

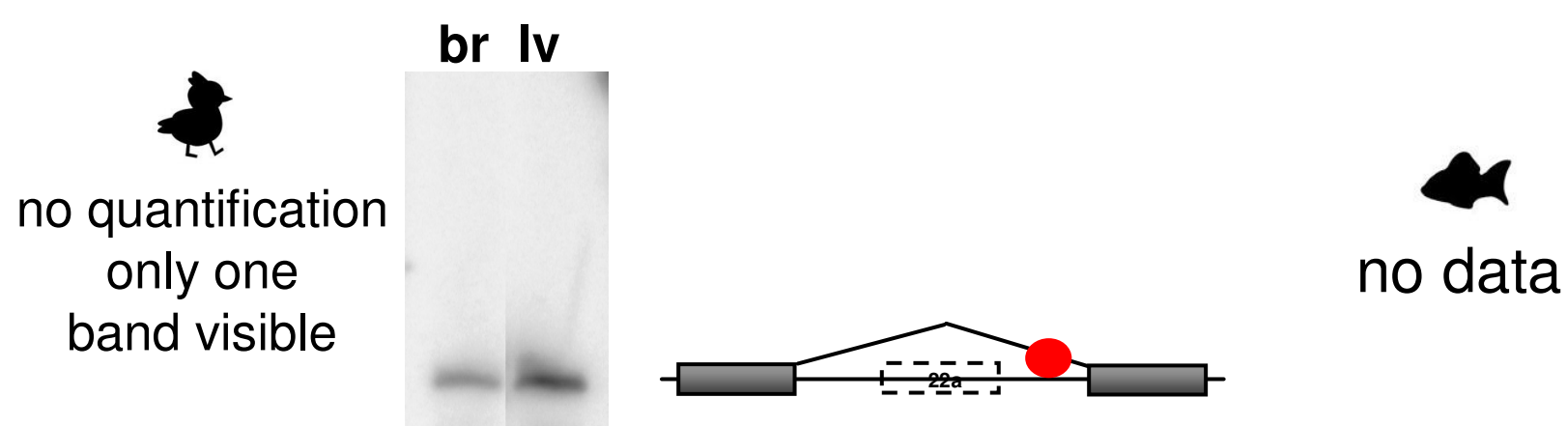

Gphn

```
          mouse niss2 cluster                                              alternative exon 7a                      mouse ness2 cluster

human   ttcatgttttcctgtcactatattcatagttgttatgtgttgtgcattagAAGCATCCATTCTACACCAGTCCTGCTGTTGTCATGGCACACGGTGAACAGCCCATCCCTGGTCTCATCAATTATTCCCATCATTCAACAGATGAA
dog     ttcatgttttcctgtcactatattcatagttgttatgtgttgtgcattagAAGCATCCATTCTACACCAGTCCAGCTGTTATCATGGCACACGGTGAGCAGCCCATTCCTGGTCTCATCAATTATTCCCATCATGCAACAGGCAgt
mouse   ttcatgtttattctcattgtattcataattgttatgtgttatacattagAAGCATCCATTCTACACCAGTCCTGCTCTTTTCATGGCAAATCATGGGCAGCCCATCCCTGGTCTCATCAGTTATTCCCATCATGCAACAGGCAgt
opossum ttcatgttttcctatcactgtattcatagctgttatgtgttgtgcattagAAGCATCCATTCTACACCAGTCCTGCTGTTATCATGGCACACGGTGAGCAGCCCATTCCTGGTCTCATCAGTTATTCCCATCACGCAACAGGCAgt
chicken ctcatatttcctgtcactgtattcatagctgttatgtgttgtgcattagAAACATCCATCCTACACCAGTTCAGCAGTTATTATGGCAAAAGGTGAACAGCCCATTCCTGGTCTCATCAGTTATTGCCATCATGCAGCAGGCAgt
         **** ****  * *** * ********  *********** * ******** ******* ********** * **  ** * ****** *   **  ******** ************* ***** ******  ** ***
```

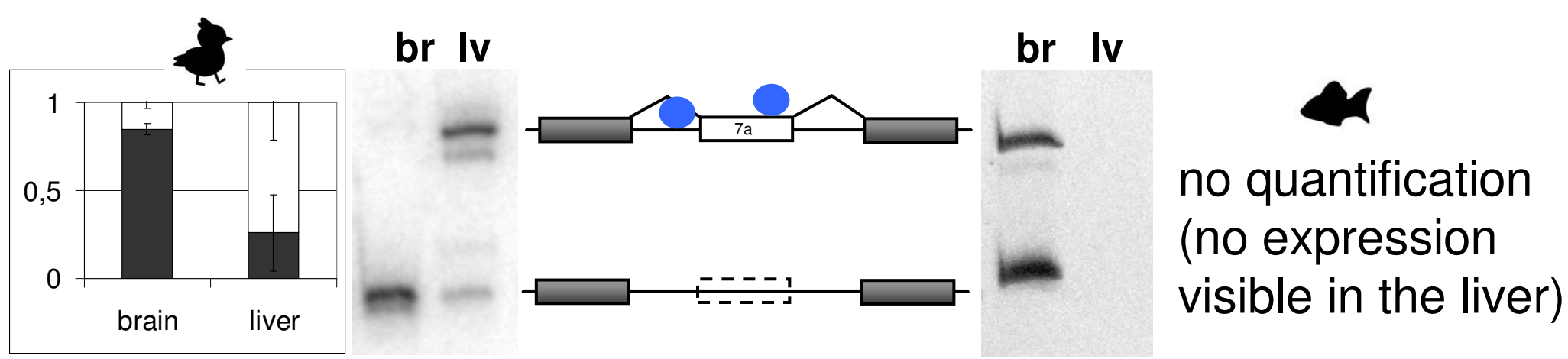

Kcnma1

```
                    5' constituitive exon                                               mouse nese1 cluster

human     agGGTTCACACCTCCAGGAATGGATAGATCCTCTCCAGATAACAGCCCAGTGCACGGGATGTTACGTCAACCATCCATCACAACTGGGGTCAACATCCCCATCATCACTGAACTAGgtagggg-tcaatgtcagctttgggggga
dog       agGGTTTACACCTCCAGGAATGGACAGATCCTCTCCAGATAACAGCCCAGTGCACGGGATGTTACGCCAGCCATCCATCACAACTGGAGTCAACATCCCCATCATTACTGAACTAGgtaggga-tgaatgtcagctttgggaaga
mouse     agGATTCACACCTCCTGGAATGGACAGATCATCACCCGACAACAGCCCAGTGCACGGGATGTTACGCCAGCCGTCCATCACAACTGGGGTCAACATTCCCATCATCACGGAACTCGgtagggggtcactgt-agctcactgcgtg
opossum   agGGTTCACACCACCAGGAATGGACAGATTATCTCCAGACAACAGTCCAGTCCACTTGCATCAGCCATCACATTCCCTCACATCTGGGGTCAGCCTCCCCATGATCACTGAGCTAGgtaaggaggtgaattt-acaacccatttt
chicken   agGCTTTACACCCCCTGGTATGGATAGGTCATCTCCAGACAACAGTCCTGTCCACGGCCTGTTACGTCAACCCTCCATTACAACAGGAGCCAACATCCCTATTATAACAGAGCTAGgtaagaagagaattt-atcctatcatttta
xenopus   agGGTTTACGCCCCCAGGAATGGAGAGATCCTCACCTGATAATAGCCCAGTGCATGGTGTTGCAAGACAGGCATCCATAACTACAGGGGCCAACATTCCCATAATTACAGAACTTGgtaagtc-tgaattt-atctacttgtgtt
zebrafish agGTTTCACACCTCCAGGGATGGATAGGTCATCTCCTGATAACAGTCCCGTGCACGGCTTAGTGCGGCAGGCATCCGTCACCACCGGATCAAACATCCCCATCATAACTGAACTGGgtgagacgcgactcc-agt-ct--gtact
          *** ** ***** ** ** ***** ** ** ** ** ** ***** ** ** *****  ******* ** ** ***** ***** ** * ****** ** ** ** ** ** ** ***          *
```

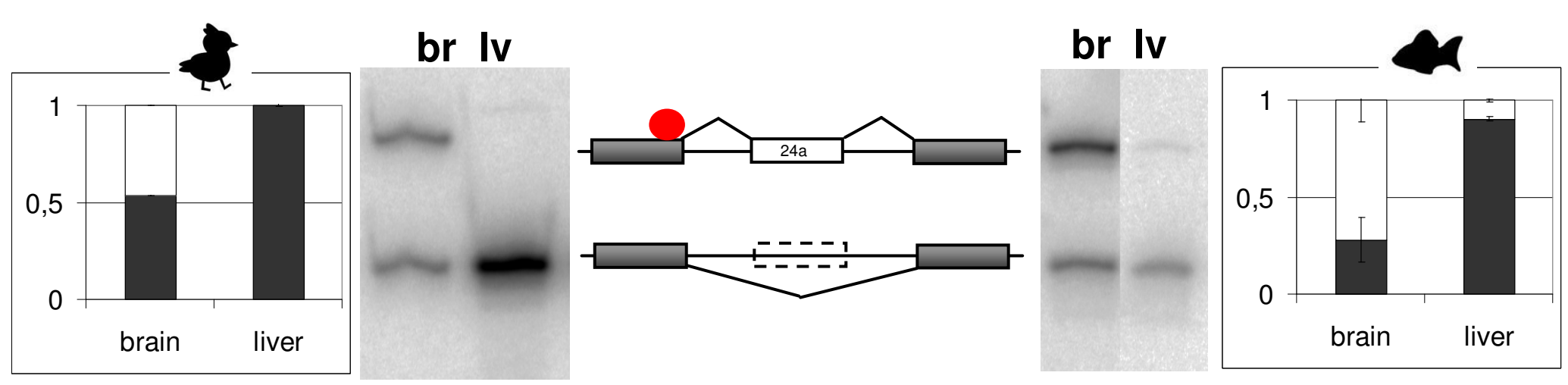

Lrrfip

```
                    mouse nisel cluster                                                                                    alternative exon 17a

human     acttccatcattcatttcactaacctgaacgatcattcttcagtttagcaatgtgctgaaaggcgcggcacgcggattcacctaaacctgtgtctttgtgatttctttagATATTAGGTTGAAAAAGCTGGTTGATGAACGGGAA
dog       accttcatcattagtttcactaacctgaatgctcactcttccatttagcattctgccgaacggtgcgctacacggattctcctcaacctctatctttctgatttctttagATATTAGGTTGAAAAAGCTTGTTGATGAACGGGAG
mouse     aacctacactccatccctcattccactaaccgatccgtctgttattttgttaggggcatgctaagaggccaggatacagattcacctatatgtctttctgatttcttcagACGTTAGGTTGAAAAAGCTCATTGATGAACGGGAA
rat       aacctacacttcatcactcattccactaaccggtctgtctgttattgtgttagtgacatgctgtgaggccaggatatagactcaccgatatgtctttctgatttcttcagATGTTAGGTTGAAAAAGCTCATTGATGAACGGGAA
opossum   tctcgctaacatacatgatcattcttttccaattttacatcatctgggccaggagggccctgtgccaattcagctctcaaacgtgtgctttctctctcttcctttctagACATTAGGCTGAAAAACCTTGTCGATGAACGAGAA
chicken   ataactaccttcatcattagtttcactaaccttaacgggtccctcttctatcagatactctgaaacgtgtggctcgctgaaatcaacactgagctttcagttttctctttcagAAAGGCTGAAAAAACTCATTGGCGAACGAGAG
xenopus   tttttttctttgttttgttcaatatttctccgtatttccatttcttttctttctcactctcctcctgttctccgcctgtaccttgcaccattgcttatcattgccgaagACATCCGGCTGAAGAAGCTGCTAGACGAAAGGGAA
zebrafish ctataacatctacggtaaattgcctcaatgtgtacgttgcaatgtctgattctgtctgtgattctgagattgatatgctgtatgtgttttgttgttgctgtaccttttagAGATCAGATTGAGGAAGCTCATGGAGGAAAGAGAG
```

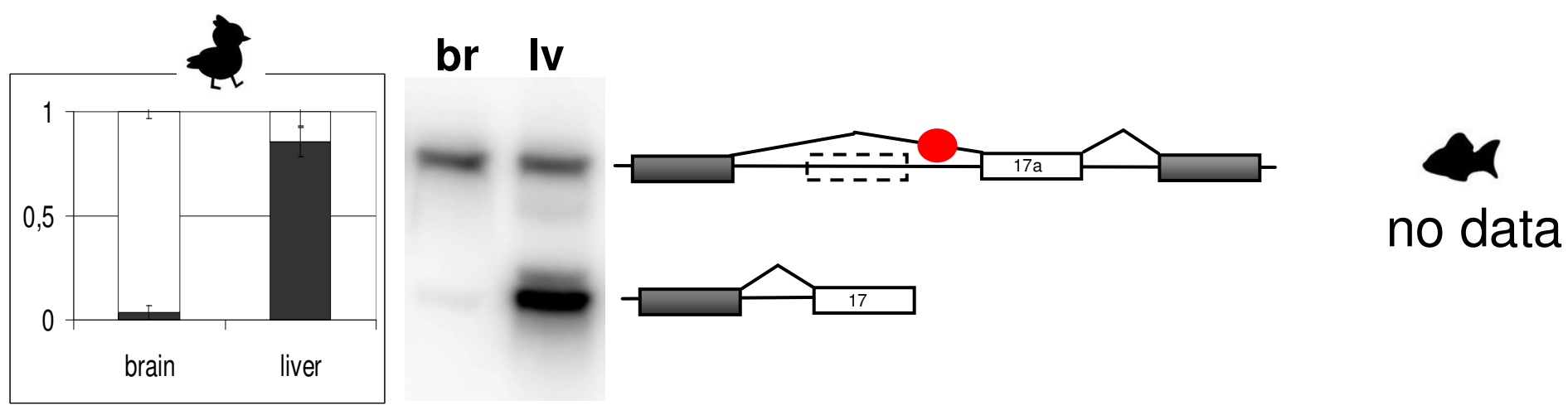

Map4

```
                                        mouse niss2 cluster                                                  alternative exon 18

human     --ccaccaccatcacc-----------ccatcccactcccccc-tcgactgacc-ctgcactgtc-tcc--cccac----------------tgtttcattgt----agACTGAGGGCGG------TG
dog       --ccaccaccaccatcaa---------ccatcccacctcctcc--ccgactgacc-ctgcactgtc-tcc--cccac----------------tgtttcattgt----agACTGAGGGCGG------TG
mouse     --tcaccaccatcacc-----------ccattccactccctcc--ccaactgacc-cttcactgtc-tcc--cccac----------------tgtttcattgt----agACTGAGGGCGG------TG
rat       --tcacctccatcacc-----------ccattccactccctcc--ccaactgacc-cttcactgtc-tcc--cccac----------------tgtttcattgt----agACTGAGGGCGG------TG
opossum   ctccatcg---tcacc-----------ccatctcacctcatcct-cctattgatc-gtccattgtc-tcctccccacccac-----------tgtttcattgt----agACTGAGGGGAG------CG
chicken   ---cagat--gtcacc-----------cc-tccggcgccccaaatcctattgacc-gctc-ctgtcgtcctccccacccgttgt--------tgtctccttgt----agGCTGAGGGCAG------CG
xenopus   ttact-cttactgaccaacctaaagaacatttgcgcggaaccct--gatttgatctgttccttgt--------cca----------------atgataca-ggt----agAC-GAAAGCCAAAATATTG
zebrafish ---atcc--------------------ccttacacacataca--cacacaaaca-aatgtttgtc-tc--tggcacccatttcttgaattcttgttt-attgtctccagGCTGAGG-AGAA-----TC
                                       *    *              *         ***          **                **         **      ** *  **
```

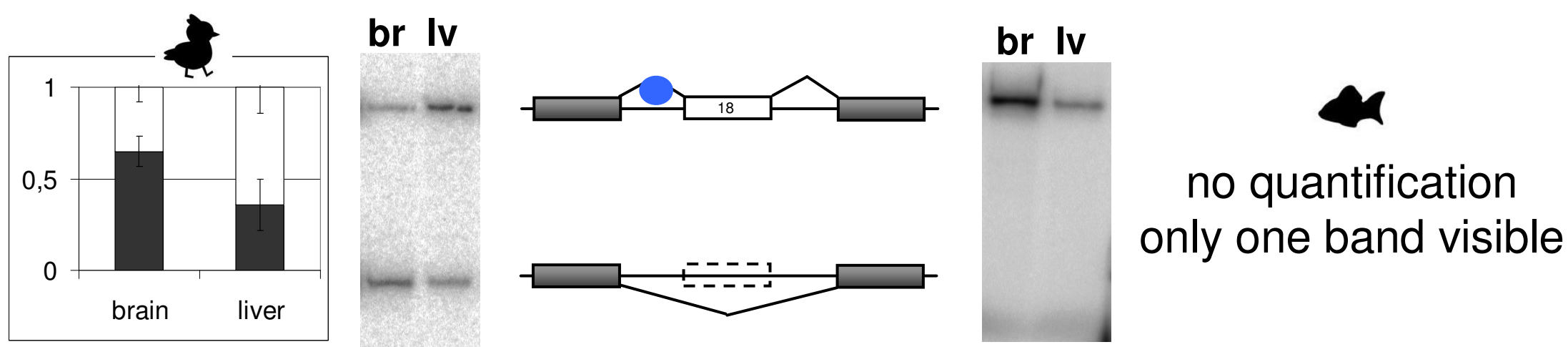

Map4k4

```
          alternative exon 22a                                               mouse ness2 cluster

human     GCCAGATCTCTTACAGCAAAGCCATTCCTCCTCCACTTCCTCCACCTCCTCCTCCCCATCCTCCAGCCAGCCGACACCCACCATGTCCCCACAGACACCCCAGGACAAGCTCACTGCTAATGAGgt
dog       GCCAGATCTCTTACAGCAAAGCCATTCCTCCTCCACTTCCTCCACCTCCTCCTCCCCATCCTCCAGCCAGCCGACACCCACCATGTCCCCACAGACACCCCAGGACAAGCTCACTACTAATGAGgt
mouse     GCCAGATCTCTTACAGCAAAGCCATTCCTCCTCCACTTCCTCCACCTCTTCCTCCCCATCCTCCAGCCAGCCGACACCCACCATGTCCCCACAGACACCCCAGGACAAGCTCACTGCTAATGAGgt
rat       GCCAGATCTCTTACAGCAAAGCCATTCCTCCTCCACTTCCTCCACCTCTTCCTCCCCATCCTCCAGCCAGCCGACACCCACCATGTCCCCACAGACACCCCAGGACAAGCTCACTGCTAATGAGgt
opossum   GCCAGATCTCTTACAGCAAAGCCATTCCTCCTCCACTTCCTCCACCTCCTCCTCCCCATCCTCCAGCCAGCCGACACCCACCATGTCCCCACAGACACCCCAGGACAAGCTCACTCCTAATGAGgt
chicken   GCCAGATCTCTTACAGCAAAGCCATTCCTCCTCCACTTCCTCCACCTCCTCTTCCCCATCCTCCAGCCAGCCGACACCCACCATGTCCCCACAGACACCCCAGGACAAGCTAACTACTAATGAGgt
xenopus   GCCAGATCTCTTACAGCAAAGCCATTCC---------TCCTCCACCACTTCTTCCCCAACCTCAAGTCAACCGACACCTACCATGTCCCCCCAGACACCCCAGGACAAGCTAACTGCTAATGAGgt
zebrafish GCCTGATCTAGTGCAGCAAAG-------------------------------------CCACTCGCCCGCCGAGGGCC-CTGGGCGGCAGTCTAGTGCGCTGGACCTGGACTCTGTGGCTGAAgt
          *** *****  * ********                                     ** *  * *  ****   *   *   *   *     *   * * ****  *    **      *** **
```

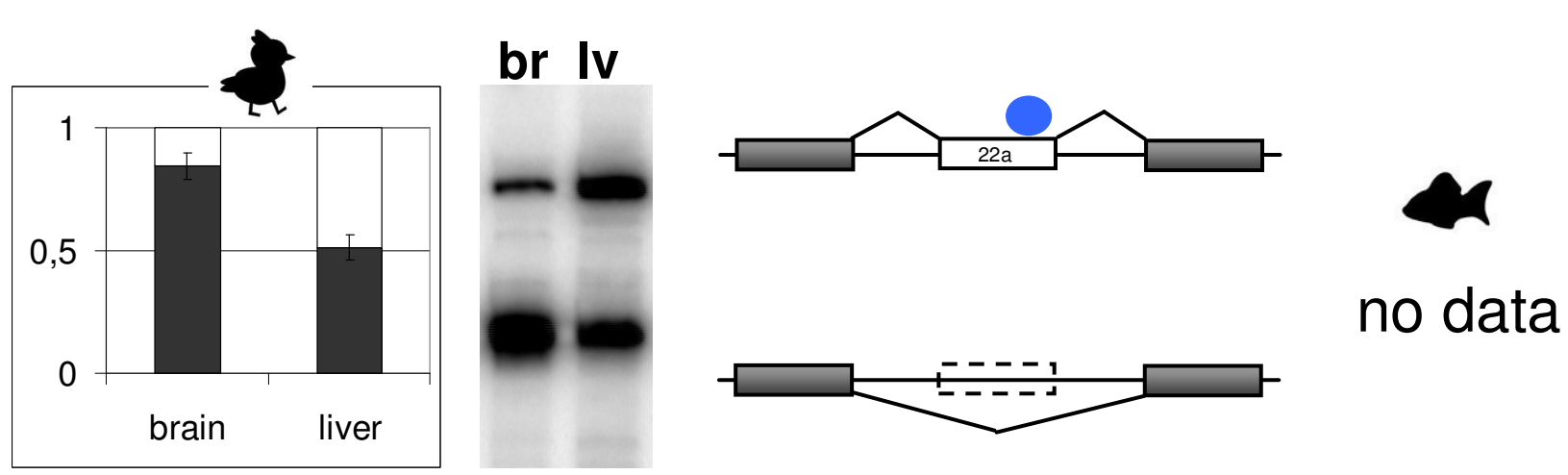

Neo1

mouse ness1 cluster *alternative exon 26*

```
human     agCTGTGATTAGTGCCCATCCCATCCATTCCCTCGATAACCCTCACCATCATTTCCACTCCAGCAGCCTCGCTTCT-CCAGCTCGCAGTCAT------CTCTACCACCCGGGCAGCCCATGGCCCATT---GGCACATCCATGTC
dog       agCTGTGATTAGTGCCCATCCCATCCATCCCCTCGATAACCCTCACCATCATTTCCACTCCAGCAGCCTCGCTTCT-CCAGCTCGCAGTCAT------CTCTACCACCCGGGCAGCCCGTGGCCCGTT---GGCACATCCCTGTC
mouse     agCTGTGATTAGTGCCCATCCCATCCATTCCCTCGATAACCCTCACCATCATTTCCACTCCAGCAGCCTCGCTTCT-CCAGCCCGCAGTCAT------CTCTACCACCCAAGCAGCCCATGGCCCATT---GGCACATCCATGTC
rat       agCTGTGATTAGTGCCCATCCCATCCATTCCCTCGATAACCCTCACCATCATTTCCACTCCAGCAGCCTCGCTTCT-CCAGCCCGCAGTCAC------CTCTACCACCCAAGCAGCCCATGGCCCATT---GGCACATCCATGTC
opossum   agCTGTGATTAGTGCCCATCCCATCCATTCCCTCGATAACCCTCACCATCATTTCCACTCCAGCAGCCTCGCCTCT-CCGACCCGCAACCAT------CTCCACCTTCAGGGCAGCCCATGGCCCATT---GGCACATCCTTGTC
chicken   agCTGTGATTAGTGCCCATCCCATCCATTCACTCGATAACCCCCACCATCATTTCCACTCCGGCAGCCTCGCTTCT-CCAACTCGCAGCTAT------CTCCATCACCAGGTCAGCCCGTGGCCAGTG---GGCACATCCATGTC
xenopus   agCGGTTGTCAGTGCCCATCCCATCCATTCCCACGATAATTCTCA---TCATTTCGCTCC--GTTCCTGGGTTCATGCTGCACG-TGTTGGGTGGCCCTCCACAGGCTGCAGGTGAGA-GGGACACTCCTGGGAATGGCAGTGT
zebrafish agCTGTGATTAGTGCTCACCCCATCCATTCCCTCGATAACCATCACCATCATTATCACTTGGGCAGCCTGGCCTCG-CCGACACGCAGCTAT------CTCCACCACCAGAGCAGCACGCGCTCCATC---GCCACCCCCATCTC
          *** **  * ***** ** ********* * * ******    **    *****  * **       *** *  **    *  * **          *** *    *         *         *  *    *
```

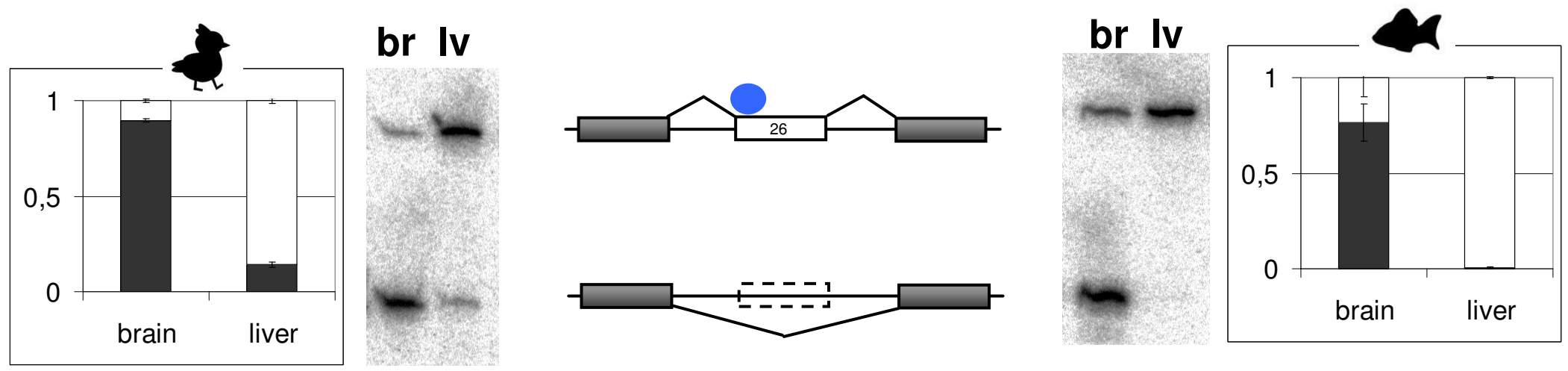

Plcl3

mouse nise2 cluster

```
human     a-taaatatat-tttaccatttattcatcacacagacactttgttgcaaacctatgca-at-ccataattatattctgtcacagt
dog       a-taaatatat-ttcgccatctactcatcacacagacactctgttgcaaacctctgcg-at-ccataact-----ctgtcacaac
mouse     g-taaatatat-ttcaccatttatccatcacacagacactgtgttgcaaacttctgcttat-ccataattatattctgtcacaat
rat       g-taaatatatatttaccatttatccatcacacagacactgtgttgcaaacttctgcttat-ccataattatattctgtcacaat
opossum   g-ccagtctagcgtcaccatctatccattacatagatattttgtactaaactcgcaca-gtcccatgagtaaat--agtcacagt
chicken   g-cagttgtat-ttttccatttattcatgtcacagataatttgggctaagcctgcgca-acaccataaca--gttctgtcacaac
xenopus   a-cctacttgt-ggggtcatgtttccatttcacataagcacttctttgtacattgaagtataccatgaaaatgtcccttcaccttt
zebrafish aataaaaaaagcaacaagtatctggatgttaaccaactatctggatgttctccatggacccacacatttcatttatctgacttgt
```

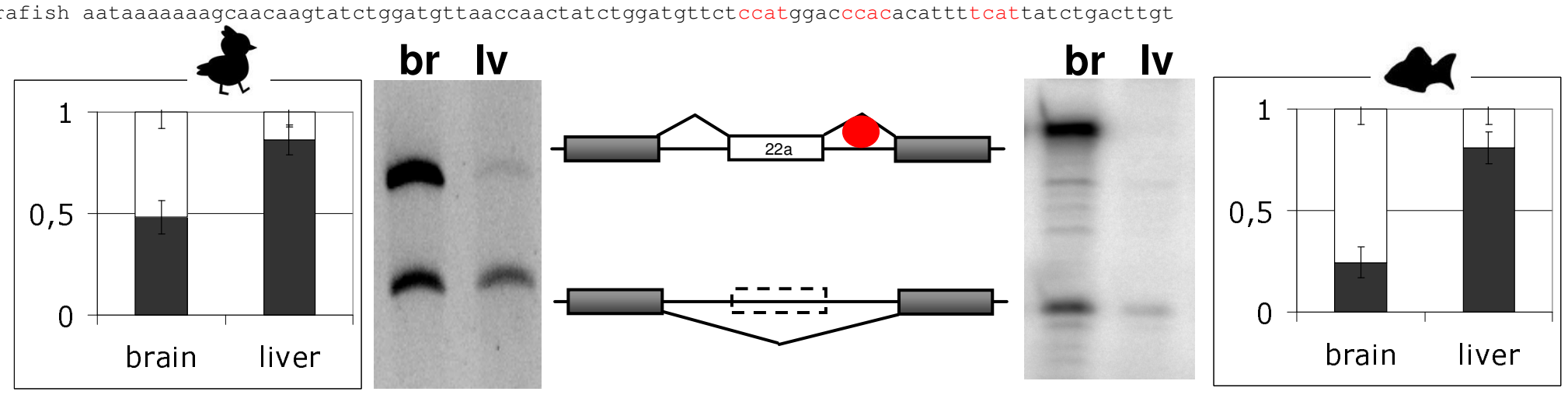

Ptprf

alternative exon 6a *mouse nise2 cluster*

```
human     agGTGGTTCACCAATCAGAGgtaagaatgctgtccgtgcctctcgccacaccacgccttgccactgccgtcacctccactccatcccgtccagtctgtcacctccccatccccatcccaacctcttcagcctcctcatctccagc
dog       agGTGGTTCACCAATCAGAGgtaagaatgctgtccgtgcatctcgccacgccacgccacgccactgccgtcaccaccactccatcccgtccagtccgtcacctcccccatccccatcctgacctctccagcctcctcatctccag
mouse     agGTGGTTCACCAATCAGAGgtaagaatgctgtccgtgcatttcgccacgccacgccacgtcacaccacgccacaccacaccactactgtcgccgtcggtcacctccactccatcccgtccagtccgtcacctccccataccccat
rat       agGTGGTTCACCAATCAGAGgtaagaatgctgtccgtgcatttcgccacgccacgccacgtcacaccacaccacaccactgctgtcgccgttggtcacctccactccatcccgtccagtccgtcacctccccatccccatcctgc
opossum   agGGGGCTCACCGATCAGAGgtaagagtgcggaccatggccccccacgccacgccacgccacgccgcctcgcctgtgtgtcactccatttactgtccacttcatccccggcgcctgctgcagcccgggtctacactcactcacc
chicken   agGTGGTACACCGATCAGAGgtaagaatgctgaaatgtgcctccagttgtcacgtcactccatctcctgtctgccttgtcccttctcctccagtgttgctcatctccagctttactctcagccctaactccgccagcgctctcgc
xenopus   agGTGGTACACCAATCAGAGgtaagaatgctgggatgtccttcagccgtcacttcacgccatattccttttgatttatccttttgaatccacagtcagcttgtcaaacctcatgtacccccttccttcagcatccacactcacaa
zebrafish agGGGGTTCGCCGATACGAGgtaagacagctggaatgtgtctcccgaaacgtctcctccattacctgtttcatcacaaccactgtcatttcctgcttgttcagtacatacacaacctttgtcgtggcattcaacctgtctgcgtt
          *** **  * ** **  *********  ** *
```

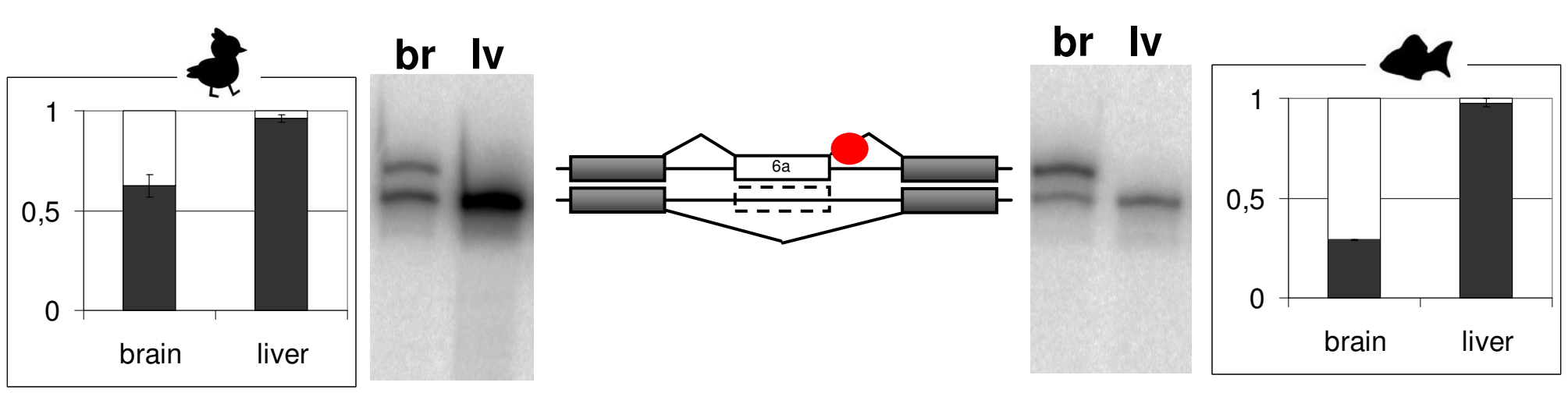

Ptprf (alt. exon 19a)

```
                    mouse nese2 cluster                    constutive exon 20

human      agGTATGCGAGACCACCCACCCATCCCCATCACCGACCTGGCGGACAACATCGAGCGCCTCAAAGCCAACGATGGCCTCAAGTTCTCCCAGGAGTATGAGgt
dog        agGTATGCGAGACCACCCGCCCATCCCCATCACTGACCTGGCCGACAACATTGAGCGCCTCAAAGCCAACGACGGCCTCAAGTTCTCCCAGGAGTACGAGgt
mouse      agGTATGCGAGACCACCCGCCCATCCCCATCACTGACCTGGCAGACAATATTGAGCGCCTCAAAGCCAACGATGGGCTCAAGTTCTCCCAGGAGTATGAGgt
rat        agGTATGCGAGACCACCCGCCCATCCCCATCACTGACCTGGCAGACAATATTGAACGTCTCAAAGCCAATGATGGGCTCAAGTTCTCCCAGGAGTATGAGgt
chicken    agGCATGCGGGACCACCCGCCCATCCCCGTGACCGACCTCGCCGACAACATCGACCGGCTGAAGGCCAACGATGGCCTCAAGTTCTCCCAGGAGTACGAGgt
xenopus    agGTATGAGAGACCACCCTCCTATTCCAATCTCTGACCTGGCAGACAACATTGAAAGACTGAAGGCTAATGATGGACTCAAGTTCTCTCAGGAATATGAGgt
zebrafish  agGAATGAGGGAACATCCTCCCATATCCGTCTCCGAACTGGCAGACCACATCGAGCGCCTCAAGGCCAACGATGCCCTCCGGTTTTCCCAAGAGTATGAGgt
           *** *** * ** ** ** ** **  *  *  * ** ** ** *** * ** **  * ** ** ** ** ** *  ***  *** ** ** ** ** *****
```

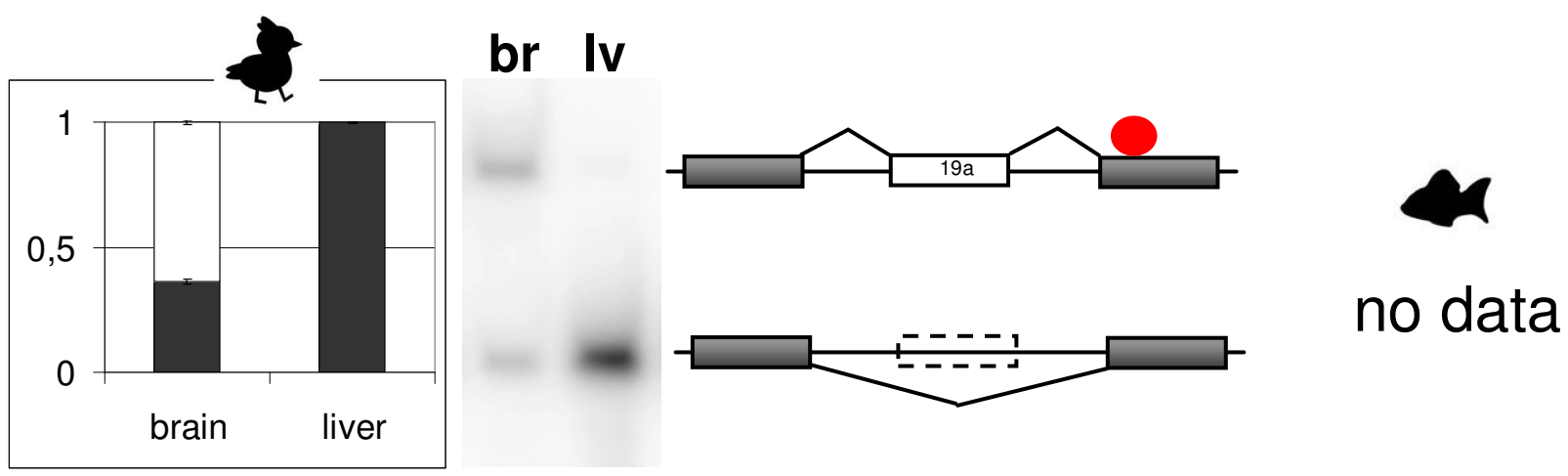

St7

```
         alternative exon 7                                                          mouse nise2 cluster

human      CAAAgtaagtcaagaacctgttgggtgccctacttctaaccaaatgagacttc---tagtggatatctggatggttgacacccccatcaccacac
dog        CAAAgtaagtcaagaacctgtcgggtgccctacttctaaccaaatgagacttcttctagcagatatctggatggttgacacccccatcaccatac
mouse      CAAAgtaagtcaagaacctgttgggcaccctacttctaaccaaacgagacttg---tagccaatatctggatggttgaca-ctccatcaccacac
rat        CAAAgtaagtcaagaacctgttgggcaccctacttctaaccaaacgagactgg---tagcagatatctggatggttgaca-cttcatcaccacac
chicken    ---------------------------------------------------------------------------------------------
zebrafish  ---------------------------------------------------------------------------------------------
           ******************** ***  **************** ******     ***   ****************** *  ******** **
```

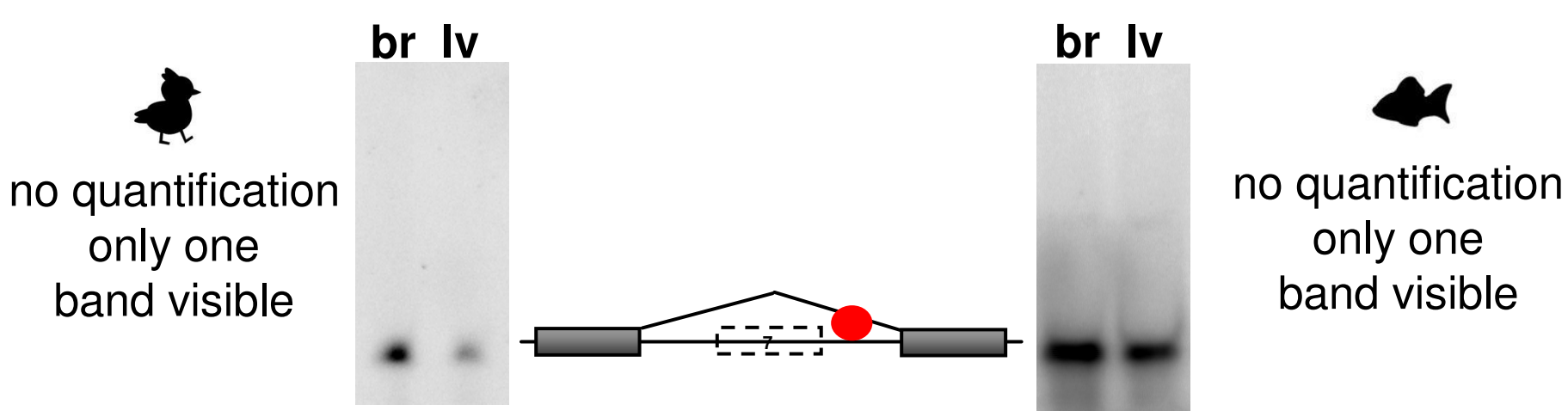

Stxbp2

```
                          mouse ness1 cluster                                   alternative exon 3

human      ctgtccatccatctg--------gacagGTGCTTATCATGGATCACCCAAGCATGCGCATCTTGTCTTCCTGCTGCAAAATGTCAGATATCCTGGCTGAGGGCATCACCAgt
dog        ctg----ttcatctg-tcc---cgacagGTACTCATCATGGACCATCCAAGCATGCGCATCTTGTCCTCCTGCTGCAAGATGTCAGACATCCTAGCTGAGGGCATTACTAgt
mouse      ataccCattcatctg--------gtcagGTCCTCATCATGGATCACCCGAGCATGCGAATCTTGTCATCCTGTTGTAAAATGTCAGATATCCTGGCGGAGGGCATCACCAgt
rat        atatccacccatctg--------gtcagGTACTCATCATGGATCATCCGAGCATGCGAATCTTGTCATCCTGTTGTAAAATGTCAGATATCTTGGCGGAGGGCATCACCAgt
opossum    att--ctagttactgctccatgctttagGTTCTCGTCATGGACCGCCCAAGCATGCGGATCCTGTCTTCCTGCTGTAAGATGTCAGACATTCTAGCAGAGGGCATCACCAgt
chicken    ctg--atggctgctg-tcc-----tcagGTGCTGGTGGTGGACCAGCTCAGCATGCGCATGCTGTCCTCCTGCTGCAAAATGACCGACATCATGACGGAGGGCATCACCAgt
xenopus    atg--ccatt---ttctcc---ccacagGTGCTTGTTGTGGATCAGCTAAGTATGCGCATGTTGTCGTCCTGCTGCAAGATGACAGATATCATGACCGAAGGGATTACTAgt
zebrafish  ------------------tatattcagGTGCTCATCGTTGATCATATCAGTATGCGCATTCTGTCCTCGTGCTGTAAAATGTCTGACATCATGGCAGAGGGTGTTACAAgt
                                   **** **  *  * ***     ** ***** **  **** ** ** ** ** *** * ** **  *  * ** **   * ** ***
```

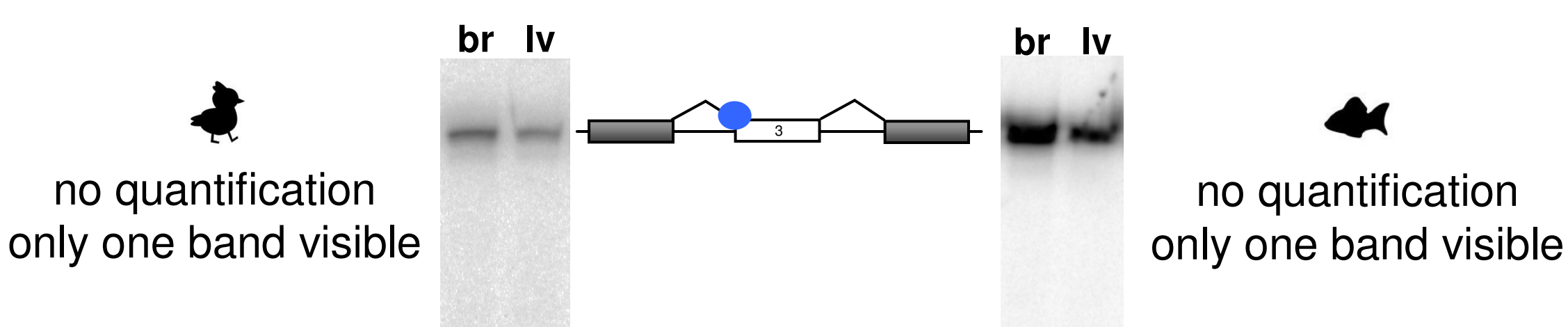

Tacc2

mouse nise3 cluster

```
human   tgcactttctcatacttttt--ga-tgttctttgttt-caaaaccatacaattggc---ccattcatgag-agggtgagtgta---------atcctctttg-tcgta---tgattaatgctaac-----------------------
dog     agcattttctcatacttct--gg-tgtcctttgtct-caaaaccatacaactgac---ccattcacgag-aggataattgca--------gatcctgtttc-tcata---tgagtgatactaac-----------------------
mouse   tgcattttctcatagtttc--ga-tgttctttgttt-caaaaccatgcagctgag---cgggtgaatgc-ggtggtcatggccacgtgtgcagcctgtgtg-tcacg---ccattagtggtaactgacctctctctctct-tctctc
rat     tgcattttctcatagtttc--ag-tgttctttgttt-caaacccatgaggctgag---cggttgaatgt-agttctcaaggccacatgtgcagccttaggcatcacg---ccattagtagtaactgttctctctctctc--tctct-
opossum cacattttctcatagtttt--ttgtgttcatcatttacaaaaaaacaaaacaaaa---caaaacaa-ac-agcacttccctactc-cccccacccccagcc-ccaaac--acatacattataactgacccattcatga-a-ccagat
chicken ctaatttggctctcctgatgcttactatga--attt---taaattgtattgaaatgttcctgtcaatgttaccactgtaatagtt-tagtagtgcacagg---ctctcagtcctagctcaggac--cctggtgcctagcacccaga-
            * **     *  *                  * *    *                *    *                        *       *           *   **
```

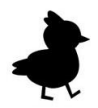

no quantification
only one band visible

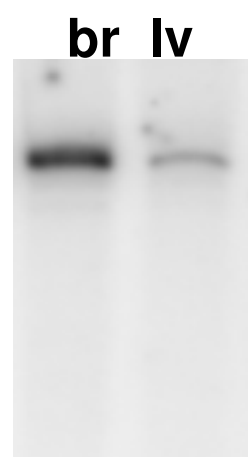

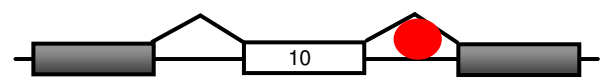

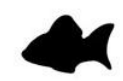

no data
