# Supplementary material for: Evolution of Nova-Dependent Splicing Regulation in the Brain
Source: PLoS Genet. 2007 Oct 12;3(10):e173. doi: 10.1371/journal.pgen.0030173 (PMC2014790; doi:10.1371/journal.pgen.0030173)
Supplement: Supplementary file 3 [file pgen.0030173.st001.doc]

Supplementary Table1a: YCAY cluster scores of orthologous sequences, calculated by the high-stringency algorithm

| **Gene symbol** | **exon** | **element** | **human** | **opossum** | **chicken** | **Xenopus** | **Zebrafish** |
| --- | --- | --- | --- | --- | --- | --- | --- |
| Agrn | 31a | NISE2 | 0.6 | 0.8 | 0.0 | 0.0 | 0.0 |
| Agrn | 31a | NISE3 | 0.0 | 0.0 | 0.0 |  | 0.0 |
| APLP2 | 14 | NISE2 | 0.8 | 0.8 | 0.6 | 0.0 | 0.0 |
| Cacna1b | 24a | NISE2 | 1.3 | 0.8 | 0.0 |  | 0.6 |
| Camk2g | 13a | NISE2 | 1.2 | 1.4 | 1.0 |  | 1.3 |
| Camk2g | 13a | NISE3 | 0.0 | 1.3 | 0.0 |  |  |
| Cask | 19 | NISE2 | 0.0 | 0.0 | 0.0 |  | 0.0 |
| Ccdc64 | 8 | NISE2 | 0.0 | 1.2 | 0.3 | 0.6 | 0.9 |
| Ccdc64 | 8 | NISE3 | 1.2 | 0.0 | 0.6 |  | 0.0 |
| Clasp1 | 9 | NESE1 | 0.3 | 0.0 | 0.0 | 0.0 | 1.3 |
| CLSTN1 | 10 | NISE2 | 0.0 | 0.0 | 0.0 |  | 0.0 |
| CLSTN1 | 10 | NISE3 | 0.0 | 0.0 | 0.0 |  | 0.0 |
| Efna5 | 3 | NISS1 | 0.3 | 0.0 | 0.0 | 0.0 | 0.0 |
| Epb4.1 | 14 | NISE2 | 1.2 | 0.6 | 0.0 |  | 1.0 |
| Epb4.1 | 14 | NISE3 | 1.3 | 1.1 | 0.3 |  | 0.0 |
| Epb41L2 | 12a | NISE2 | 0.6 | 0.8 | 0.0 | 1.0 |  |
| Epb41L2 | 12a | NISE3 | 0.6 | 1.2 | 0.6 | 0.6 |  |
| Golga4 | 22a | NISE2 | 0.0 | 0.0 | 0.0 |  |  |
| Golga4 | 22a | NISE3 | 0.0 | 1.2 | 0.0 |  |  |
| Gphn | 7a | NESS2 | 0.3 | 0.3 | 0.3 |  | 1.1 |
| Gphn | 7a | NISS1 | 0.0 | 0.0 | 0.0 | 0.0 | 0.0 |
| Gphn | 7a | NISS2 | 0.0 | 0.0 | 0.0 |  | 0.0 |
| Gpr45 | 2 | NESS | 2.2 |  |  |  |  |
| Grik2 | 15a | NESS2 | 1.9 | 0.0 | 0.0 |  |  |
| Grik2 | 15a | NISS1 | 0.0 | 0.0 | 0.0 | 0.0 | 0.0 |
| Grin1 | 19 | NISE2 | 0.0 | 0.0 |  | 0.0 |  |
| Idh3b | 12 | NESE2 | 0.0 | 0.3 | 0.0 |  | 0.3 |
| Igsf4a | 8 | NESS2 | 2.3 | 1.7 | 1.6 |  | 1.5 |
| Kcnma1 | 24a | NESE1 | 0.9 | 0.3 | 0.0 | 0.0 | 0.0 |
| Lrp1b | 79 | NISE2 | 0.6 | 0.9 | 1.1 | 0.0 | 0.0 |
| Map4k4 | 23a | NESS2 | 0.3 | 0.3 | 0.3 | 0.0 | 0.0 |
| Map4k4 | 23a | NISS1 | 1.4 | 1.1 | 1.1 | 0.3 |  |
| Mapk9 | 6a | NISS2 | 0.3 | 0.3 | 0.3 | 0.0 | 0.0 |
| Ncdn | 1 | (NISS3) | 0.0 | 0.0 |  |  |  |
| Ncdn | 1 | NESS2 | 0.0 | 0.0 |  |  |  |
| Neo1 | 26 | NESS1 | 1.4 | 1.4 | 1.5 | 1.3 | 1.6 |
| Nova1 | 4 | NESS | 0.6 | 0.6 | 0.6 | 0.6 |  |
| Plcl3 | 22a | NISE2/3 | 0.3 | 0.3 | 0.6 | 0.6 | 0.6 |
| Ptprf | 19a | NESE2 | 1.3 | 0.9 | 0.0 | 0.0 | 0.0 |
| Ptprf | 19a | NISE3 | 0.6 | 1.1 | 0.0 | 0.3 | 0.0 |
| Rap1ga1 | 27 | NISS1 | 0.0 | 0.3 |  |  | 0.6 |
| Skip | 10 | NISE2 | 0.3 | 0.0 | 0.0 |  |  |
| Skip | 10 | NISE3 | 0.6 | 0.0 | 0.0 |  |  |
| Spna2 | 4 | NISE2 | 0.0 | 0.0 | 0.6 |  | 0.9 |
| St7 | 7 | NISE2 | 0.6 |  |  |  |  |
| Stxbp2 | 3 | NESS | 0.6 | 0.0 | 0.0 | 0.0 | 0.0 |
| Tacc2 | 10 | NISE3 | 0.3 | 1.2 | 0.0 |  |  |
| Tpm3 | 6 | (NISS2) | 0.0 | 0.0 | 0.0 |  | 0.0 |
| Tpm3 | 6 | (NISS3) | 0.0 | 0.0 | 0.0 | 0.0 |  |

Supplementary Table1b: YCAY cluster scores of orthologous sequences, calculated by the low-stringency algorithm

| **Gene symbol** | **exon** | **element** | **human** | **opossum** | **chicken** | **Xenopus** | **Zebrafish** |
| --- | --- | --- | --- | --- | --- | --- | --- |
| Agrn | 31a | NISE2 | 0.9 | 1.4 | 0.3 | 0.3 | 0.3 |
| Agrn | 31a | NISE3 | 0.0 | 0.0 | 0.0 |  | 0.0 |
| APLP2 | 14 | NISE2 | 1.4 | 1.4 | 1.1 | 0.0 | 0.0 |
| Cacna1b | 24a | NISE2 | 1.6 | 1.1 | 0.9 | 1.2 | 0.6 |
| Camk2g | 13a | NISE2 | 1.3 | 1.7 | 1.3 | 1.7 | 1.4 |
| Camk2g | 13a | NISE3 | 0.8 | 1.4 | 1.3 | 1.0 |  |
| Cask | 19 | NISE2 | 0.6 | 0.0 | 0.0 |  | 0.0 |
| Ccdc64 | 8 | NISE2 | 0.0 | 1.3 | 1.3 | 1.3 | 1.6 |
| Ccdc64 | 8 | NISE3 | 1.5 | 0.0 | 1.3 | 0.0 | 0.6 |
| Clasp1 | 9 | NESE1 | 0.8 | 0.3 | 0.0 | 0.6 | 1.4 |
| CLSTN1 | 10 | NISE2 | 0.0 | 0.0 | 0.0 |  | 0.0 |
| CLSTN1 | 10 | NISE3 | 0.0 | 0.0 | 0.0 | 0.0 | 0.0 |
| Efna5 | 3 | NISS1 | 0.9 | 0.8 | 0.9 | 0.0 |  |
| Epb4.1 | 14 | NISE2 | 1.5 | 1.1 | 0.6 | 1.2 | 1.5 |
| Epb4.1 | 14 | NISE3 | 1.6 | 1.4 | 1.6 | 1.1 | 1.5 |
| Epb41L2 | 12a | NISE2 | 1.2 | 1.1 | 0.0 | 1.4 |  |
| Epb41L2 | 12a | NISE3 | 1.1 | 1.6 | 0.6 | 1.1 |  |
| Golga4 | 22a | NISE2 | 0.0 | 0.0 |  |  |  |
| Golga4 | 22a | NISE3 | 0.6 | 1.3 | 0.0 |  |  |
| Gphn | 7a | NESS2 | 0.9 | 0.9 | 0.9 |  | 1.4 |
| Gphn | 7a | NISS1 | 0.0 | 0.0 | 1.2 | 0.0 | 0.0 |
| Gphn | 7a | NISS2 | 0.6 | 0.6 | 0.6 |  | 0.0 |
| Gpr45 | 2 | NESS | 2.3 |  |  |  |  |
| Grik2 | 15a | NESS2 | 2.1 | 0.0 | 0.0 |  |  |
| Grik2 | 15a | NISS1 | 0.0 | 0.0 | 0.0 | 0.0 | 0.0 |
| Grin1 | 19 | NISE2 | 1.1 | 1.3 | 1.1 | 0.6 | 1.3 |
| Idh3b | 12 | NESE2 | 1.0 | 1.2 | 0.0 |  | 0.6 |
| Igsf4a | 8 | NESS2 | 2.4 | 1.9 | 1.9 |  | 1.8 |
| Kcnma1 | 24a | NESE1 | 1.4 | 1.1 | 0.3 | 0.0 | 0.8 |
| Lrp1b | 79 | NISE2 | 1.0 | 1.1 | 1.3 | 0.6 | 0.0 |
| Map4k4 | 23a | NESS2 | 1.0 | 1.0 | 0.6 | 0.0 |  |
| Map4k4 | 23a | NISS1 | 1.7 | 1.4 | 1.4 | 1.0 |  |
| Mapk9 | 6a | NISS2 | 1.1 | 1.0 | 1.0 | 0.0 | 0.0 |
| Ncdn | 1 | (NISS3) | 0.0 | 0.0 |  |  |  |
| Ncdn | 1 | NESS2 | 0.6 | 0.6 |  |  |  |
| Neo1 | 26 | NESS1 | 1.7 | 1.7 | 1.8 | 1.6 | 1.9 |
| Nova1 | 4 | NESS | 1.0 | 1.0 | 1.0 | 1.1 |  |
| Plcl3 | 22a | NISE2/3 | 0.6 | 0.6 | 0.9 | 0.9 | 0.9 |
| Ptprf | 19a | NESE2 | 1.5 | 1.1 | 0.0 | 0.0 | 0.0 |
| Ptprf | 19a | NISE3 | 1.0 | 1.3 | 0.9 | 0.8 | 0.0 |
| Rap1ga1 | 27 | NISS1 | 0.3 | 0.8 |  |  | 0.9 |
| Skip | 10 | NISE2 | 1.2 | 0.0 | 0.0 | 0.0 |  |
| Skip | 10 | NISE3 | 0.9 | 1.0 | 0.0 | 0.0 |  |
| Spna2 | 4 | NISE2 | 0.0 | 0.0 | 1.1 | 0.0 | 1.7 |
| St7 | 7 | NISE2 | 0.9 |  |  |  |  |
| Stxbp2 | 3 | NESS | 1.3 | 0.0 | 0.0 | 0.0 | 0.0 |
| Tacc2 | 10 | NISE3 | 0.8 | 1.5 | 0.0 | 0.6 |  |
| Tpm3 | 6 | (NISS2) | 0.6 | 0.0 | 0.0 |  | 0.0 |
| Tpm3 | 6 | (NISS3) | 0.8 | 0.6 | 0.0 | 0.0 | 0.0 |
