# Supplementary material for: Evolution of Nova-Dependent Splicing Regulation in the Brain
Source: PLoS Genet. 2007 Oct 12;3(10):e173. doi: 10.1371/journal.pgen.0030173 (PMC2014790; doi:10.1371/journal.pgen.0030173)
Supplement: Supplementary file 4 [file pgen.0030173.st002.doc]

Supplementary Table 2: Scores of YCAY clusters in RNA maps of all validated Nova-regulated exons included in this study, together with exon inclusion levels.

| **Gene symbol** | **exon** | **element** | **YCAY cluster score** | **ΔI (Nova wt/ko)** |
| --- | --- | --- | --- | --- |
| Agrn | 31a | NISE3 | 0.8 | 0.24 |
| Agrn | 31a | NISE2 | 1.1 | 0.24 |
| Ank3 | 31 | NISS2 | 1.6 | -0.33 |
| Ap1gbp1 | 6a | NISS2 | 1.5 | -0.34 |
| APLP2 | 14 | NISE2 | 1.1 | 0.54 |
| Atp13a4 | 7 | NESS2 | 1.4 | -0.05 |
| Atp2b1 | 34 | NISE3 | 0.0 | 0.7 |
| Atxn7l4 | 3 | NESS1 | 2.4 | -0.10 |
| Bcas1 | 9 | NISE2 | 1.2 | 0.80 |
| BRD9 | 5 | NISE1 | 1.5 | 0.35 |
| Cacna1b | 24a | NISE2 | 1.0 | 0.46 |
| Camk2g | 13a | NISE3 | 1.1 | 0.28 |
| Camk2g | 13a | NISE2 | 1.3 | 0.28 |
| Cask | 19 | NISE3 | 0.3 | 0.36 |
| Cask | 19 | NISE2 | 0.6 | 0.36 |
| Ccdc64 | 8 | NISE2 | 1.3 | 0.19 |
| Ccdc64 | 8 | NISE3 | 1.3 | 0.19 |
| Ccdc9 | 1a | NISE2 | 1.6 | 0.36 |
| Clasp1 | 9 | NESE1 | 0.8 | 0.44 |
| CLSTN1 | 10 | NISE2 | 1.4 | 0.40 |
| CLSTN1 | 10 | NISE3 | 1.5 | 0.40 |
| CP110 | 14 | NISE2 | 1.3 | 0.42 |
| CP110 | 14a | NISS1 | 1.3 | -0.42 |
| Dab1 | 7b | NISS2 | 1.3 | -0.82 |
| Dab1 | 7c | NISS2 | 1.6 | -0.82 |
| Ddr1 | 13a | (NISE0) | 1.1 | 0.14 |
| Ddr1 | 13a | NISE1 | 1.5 | 0.14 |
| Dnajb5 | 1a | NISE1 | 0.9 | 0.12 |
| Efna5 | 3 | NISS1 | 0.9 | -0.17 |
| Epb4.1 | 14 | NISE3 | 0.8 | 0.14 |
| Epb4.1 | 14 | NISE2 | 1.5 | 0.14 |
| Epb41L1 | 16 | NESS2 | 1.4 | -0.14 |
| Epb41L2 | 12a | NISE3 | 0.8 | 0.30 |
| Epb41L2 | 12a | NISE2 | 1.3 | 0.30 |
| Gabrg2 | 9 | NISE3 | 1.1 | 0.12 |
| Glra2 | 3a | NISE1 | 1.4 | 0.1 |
| Golga4 | 22a | NISE3 | 0.6 | 0.15 |
| Golga4 | 22a | NISE2 | 1.3 | 0.15 |
| Gphn | 7a | NESS2 | 0.6 | -0.69 |
| Gphn | 7a | NISS2 | 0.6 | -0.69 |
| Gphn | 7a | NISS1 | 1.1 | -0.69 |
| Gpr45 | 2 | NESS | 2.3 | -0.81 |
| Grik2 | 15a | NISS1 | 1.2 | -0.22 |
| Grik2 | 15a | NESS2 | 2.1 | -0.22 |
| Grin1 | 19 | NISE2 | 1.4 | 0.32 |
| Idh3b | 12 | NESE2 | 1.1 | 0.53 |
| IGgsf4B | 2 | NISE2 | 1.9 | 0.22 |
| Igsf4a | 8 | NESS2 | 2.3 | -0.23 |
| IGSF4B | 2 | NISE3 | 1.6 | 0.22 |
| ITGA3 | 25 | NISS1 | 1.0 | -0.08 |
| Kcnc2 | 11a | NISE3 | 1.2 | 0.12 |
| Kcnma1 | 24a | NESE1 | 1.5 | 0.4 |
| Lrp1b | 79 | NISE2 | 1.6 | 0.57 |
| Lrrfip1 | 17a | NISE1 | 1.0 | 0.69 |
| Map4 | 18 | NISS2 | 1.8 | -0.80 |
| Map4k4 | 23a | NESS2 | 0.6 | -0.15 |
| Map4k4 | 23a | NISS1 | 1.6 | -0.15 |
| Mapk9 | 6a | NISS2 | 1.0 | -0.43 |
| Ncdn | 1 | NESS2 | 0.6 | -0.26 |
| Ncdn | 1 | (NISS3) | 0.6 | -0.26 |
| Neo1 | 26 | NESS1 | 1.7 | -0.69 |
| Nova1 | 4 | NESS | 1.0 | -0.3 |
| Pdlim5 | 6 | NESS1 | 1.9 | -0.22 |
| Plcl3 | 22a | NISE2/3 | 1.1 | 0.46 |
| Ptprf | 19a | NESE2 | 1.1 | 0.61 |
| Ptprf | 19a | NISE2 | 0.3 | 0.61 |
| Ptprf | 19a | NISE3 | 0.8 | 0.61 |
| Ptprf | 6a | NISE2 | 2.0 | 0.58 |
| Rap1ga1 | 27 | NISS1 | 1.2 | -0.33 |
| Sh2bpsm1 | 9 | NISS2 | 1.4 | -0.58 |
| Skip | 10 | NISE3 | 1.0 | 0.26 |
| Skip | 10 | NISE2 | 1.1 | 0.26 |
| Smarcc2 | 16a | NISE1 | 1.4 | 0.24 |
| Spna2 | 4 | NISE2 | 0.6 | 0.46 |
| St7 | 7 | NISE2 | 0.9 | 0.63 |
| Stxbp2 | 3 | NESS | 1.3 | -0.14 |
| Tacc2 | 10 | NISE3 | 1.0 | 0.38 |
| Tpm3 | 6 | (NISS3) | 0.9 | -0.43 |
| Tpm3 | 6 | (NISS2) | 1.0 | -0.43 |
