# Supplementary material for: Evolution of Nova-Dependent Splicing Regulation in the Brain
Source: PLoS Genet. 2007 Oct 12;3(10):e173. doi: 10.1371/journal.pgen.0030173 (PMC2014790; doi:10.1371/journal.pgen.0030173)
Supplement: Supplementary file 5 [file pgen.0030173.st003.doc]

Supplementary Table 3: Relative expression levels of Nova regulated genes in mouse brain and liver.

| **Gene symbol** | **element** | **exon** | **YCAY** | **ΔI (Nova wt/ko)** | **brain median** | **liver** | **brain/liver** |
| --- | --- | --- | --- | --- | --- | --- | --- |
| Agrn | NISE2 | 31a | 1.1 | 0.24 | 1105.0 | 380.9 | 2.9 |
| Agrn | NISE3 | 31a | 0.8 | 0.24 | 1105.0 | 380.9 | 2.9 |
| Ank3 | NISS2 | 31 | 1.6 | -0.33 | 19028.0 | 930.1 | 20.5 |
| Ap1gbp1 | NISS2 | 6a | 1.5 | -0.34 | 295.6 | 168.4 | 1.8 |
| APLP2 | NISE2 | 14 | 1.1 | 0.54 | 24596.9 | 10916.8 | 2.3 |
| Atp13a4 | NESS2 | 7 | 1.4 | -0.05 | 79.2 | 74.3 | 1.1 |
| Atp2b1 | NISE3 | 34 | 0.0 | 0.7 | 64.3 | 48.9 | 1.3 |
| Atxn7l4 | NESS1 | 3 | 2.4 | -0.10 | 747.7 | 526.5 | 1.4 |
| Bcas1 | NISE2 | 9 | 1.2 | 0.80 | 2023.1 | 69.0 | 29.3 |
| BRD9 | NISE1 | 5 | 1.5 | 0.35 | no data about expression |  |  |
| Cacna1b | NISE2 | 24a | 1.0 | 0.46 | 35.2 | 35.6 | 1.0 |
| Camk2g | NISE2 | 13a | 1.3 | 0.28 | 13250.8 | 589.1 | 22.5 |
| Camk2g | NISE3 | 13a | 1.1 | 0.28 | 13250.8 | 589.1 | 22.5 |
| Cask | NISE2 | 19 | 0.6 | 0.36 | 401.9 | 370.9 | 1.1 |
| Cask | NISE3 | 19 | 0.3 | 0.36 | 401.9 | 370.9 | 1.1 |
| Ccdc64 | NISE2 | 8 | 1.3 | 0.19 | expressed in liver, data from ArrayExpress |  |  |
| Ccdc64 | NISE3 | 8 | 1.3 | 0.19 | expressed in liver, data from ArrayExpress |  |  |
| Ccdc9 | NISE2 | 1a | 1.6 | 0.36 | 1189.3 | 2217.7 | 0.5 |
| Clasp1 | NESE1 | 9 | 0.8 | 0.44 | 5346.5 | 401.4 | 13.3 |
| CLSTN1 | NISE2 | 10 | 1.4 | 0.40 | 3252.2 | 669.3 | 4.9 |
| CLSTN1 | NISE3 | 10 | 1.5 | 0.40 | 3252.2 | 669.3 | 4.9 |
| CP110 | NISE2 | 14 | 1.3 | 0.42 | no data about expression |  |  |
| CP110 | NISS1 | 14a | 1.3 | -0.42 | no data about expression |  |  |
| Dab1 | NISS2 | 7b | 1.3 | -0.82 | 168.9 | 193.5 | 0.9 |
| Dab1 | NISS2 | 7c | 1.6 | -0.82 | 168.9 | 193.5 | 0.9 |
| Ddr1 | (NISE0) | 13a | 1.1 | 0.14 | 47.1 | 31.0 | 1.5 |
| Ddr1 | NISE1 | 13a | 1.5 | 0.14 | 47.1 | 31.0 | 1.5 |
| Dnajb5 | NISE1 | 1a | 0.9 | 0.12 | 206.0 | 65.4 | 3.2 |
| Efna5 | NISS1 | 3 | 0.9 | -0.17 | 164.2 | 183.2 | 0.9 |
| Epb4.1 | NISE2 | 14 | 1.5 | 0.14 | expressed in liver, data from splicing microarray |  |  |
| Epb4.1 | NISE3 | 14 | 0.8 | 0.14 | expressed in liver, data from splicing microarray |  |  |
| Epb41L1 | NESS2 | 16 | 1.4 | -0.14 | 1155.4 | 122.6 | 9.4 |
| Epb41L2 | NISE2 | 12a | 1.3 | 0.30 | expressed in liver, data from splicing microarray |  |  |
| Epb41L2 | NISE3 | 12a | 0.8 | 0.30 | expressed in liver, data from splicing microarray |  |  |
| Gabrg2 | NISE3 | 9 | 1.1 | 0.12 | 3916.3 | 183.8 | 21.3 |
| Glra2 | NISE1 | 3a | 1.4 | 0.1 | 132.0 | 68.6 | 1.9 |
| Golga4 | NISE2 | 22a | 1.3 | 0.15 | 1637.6 | 806.4 | 2.0 |
| Golga4 | NISE3 | 22a | 0.6 | 0.15 | 1637.6 | 806.4 | 2.0 |
| Gphn | NESS2 | 7a | 0.6 | -0.69 | 3201.5 | 3405.3 | 0.9 |
| Gphn | NISS1 | 7a | 1.1 | -0.69 | 3201.5 | 3405.3 | 0.9 |
| Gphn | NISS2 | 7a | 0.6 | -0.69 | 3201.5 | 3405.3 | 0.9 |
| Gpr45 | NESS | 2 | 2.3 | -0.81 | 52.7 | 69.2 | 0.8 |
| Grik2 | NESS2 | 15a | 2.1 | -0.22 | 41.0 | 40.1 | 1.0 |
| Grik2 | NISS1 | 15a | 1.2 | -0.22 | 41.0 | 40.1 | 1.0 |
| Grin1 | NISE2 | 19 | 1.4 | 0.32 | 172.9 | 103.4 | 1.7 |
| Idh3b | NESE2 | 12 | 1.1 | 0.53 | 9516.1 | 7355.9 | 1.3 |
| IGgsf4B | NISE2 | 2 | 1.9 | 0.22 | 2016.9 | 512.8 | 3.9 |
| Igsf4a | NESS2 | 8 | 2.3 | -0.23 | 10355.1 | 726.1 | 14.3 |
| IGSF4B | NISE3 | 2 | 1.6 | 0.22 | 2016.9 | 512.8 | 3.9 |
| ITGA3 | NISS1 | 25 | 1.0 | -0.08 | 187.9 | 206.3 | 0.9 |
| Kcnc2 | NISE3 | 11a | 1.2 | 0.12 | 112.8 | 156.5 | 0.7 |
| Kcnma1 | NESE1 | 24a | 1.5 | 0.4 | 2910.7 | 625.5 | 4.7 |
| Lrp1b | NISE2 | 79 | 1.6 | 0.57 | 46.4 | 48.9 | 0.9 |
| Lrrfip1 | NISE1 | 17a | 1.0 | 0.69 | 335.5 | 85.9 | 3.9 |
| Map4 | NISS2 | 18 | 1.8 | -0.80 | expressed in liver, data from ArrayExpress |  |  |
| Map4k4 | NESS2 | 23a | 0.6 | -0.15 | 260.6 | 309.2 | 0.8 |
| Map4k4 | NISS1 | 23a | 1.6 | -0.15 | 260.6 | 309.2 | 0.8 |
| Mapk9 | NISS2 | 6a | 1.0 | -0.43 | 4096.2 | 343.0 | 11.9 |
| Ncdn | (NISS3) | 1 | 0.6 | -0.26 | 26340.2 | 457.0 | 57.6 |
| Ncdn | NESS2 | 1 | 0.6 | -0.26 | 26340.2 | 457.0 | 57.6 |
| Neo1 | NESS1 | 26 | 1.7 | -0.69 | 1189.5 | 959.4 | 1.2 |
| Nova1 | NESS | 4 | 1.0 | -0.3 | 261.9 | 251.4 | 1.0 |
| Pdlim5 | NESS1 | 6 | 1.9 | -0.22 | 978.8 | 1028.8 | 1.0 |
| Plcl3 | NISE2/3 | 22a | 1.1 | 0.46 | 35.4 | 40.9 | 0.9 |
| Ptprf | NESE2 | 19a | 1.1 | 0.61 | 935.8 | 1746.4 | 0.5 |
| Ptprf | NISE2 | 19a | 0.3 | 0.61 | 935.8 | 1746.4 | 0.5 |
| Ptprf | NISE2 | 6a | 2.0 | 0.58 | 935.8 | 1746.4 | 0.5 |
| Ptprf | NISE3 | 19a | 0.8 | 0.61 | 935.8 | 1746.4 | 0.5 |
| Rap1ga1 | NISS1 | 27 | 1.2 | -0.33 | 147.1 | 157.2 | 0.9 |
| Sh2bpsm1 | NISS2 | 9 | 1.4 | -0.58 | 1419.9 | 1001.5 | 1.4 |
| Skip | NISE2 | 10 | 1.1 | 0.26 | no data about expression |  |  |
| Skip | NISE3 | 10 | 1.0 | 0.26 | no data about expression |  |  |
| Smarcc2 | NISE1 | 16a | 1.4 | 0.24 | 43.1 | 44.2 | 1.0 |
| Spna2 | NISE2 | 4 | 0.6 | 0.46 | 14239.5 | 1290.5 | 11.0 |
| St7 | NISE2 | 7 | 0.9 | 0.63 | 203.2 | 136.9 | 1.5 |
| Stxbp2 | NESS | 3 | 1.3 | -0.14 | 147.6 | 180.4 | 0.8 |
| Tacc2 | NISE3 | 10 | 1.0 | 0.38 | 990.2 | 71.0 | 13.9 |
| Tpm3 | (NISS2) | 6 | 1.0 | -0.43 | 6374.6 | 5204.8 | 1.2 |
| Tpm3 | (NISS3) | 6 | 0.9 | -0.43 | 6374.6 | 5204.8 | 1.2 |
