# Supplementary material for: Evolution of Nova-Dependent Splicing Regulation in the Brain
Source: PLoS Genet. 2007 Oct 12;3(10):e173. doi: 10.1371/journal.pgen.0030173 (PMC2014790; doi:10.1371/journal.pgen.0030173)
Supplement: Supplementary file 6 [file pgen.0030173.st004.doc]

Supplementary Table 4: Exon length conservation of 88 mouse Nova alternative exons in different vertebrate species.

|  |  |  | **exon length (in nucleotides)** | | | | | | |  |
| --- | --- | --- | --- | --- | --- | --- | --- | --- | --- | --- |
| **gene** | **exon** | **exon type** | **human** | **mouse** | **rat** | **opossum** | **chicken** | **xenopus** | **zebrafish** | **%EST** |
| **Agrn** | **31a** | internal | 24 | 24 | 24 | 24 | 24 | 24 | 24 | minor |
| **Agrn** | **31b** | internal | 33 | 33 | unavail. | 33 | 33 | 33 | 33 | minor |
| **Ank3** | **17** | var5 | 667 | 679 | 679 | 679 | 673 | 682 | 688 | major |
| **Ank3** | **31** | internal | 103 | 103 | 103 | 103 | 103 | 103 | 103 | major |
| **Ap1gbp1** | **6a** | internal | 303 | 303 | 303 | 303 | absent | absent | absent | minor |
| **Aplp2** | **12a** | internal | 36 | 36 | 36 | 36 | 36 | 36 | 36 | minor |
| **Arhgap21** | **8** | internal | unavail. | unavail. | unavail. | unavail. | unavail. | unavail. | unavail. | minor |
| **Atp13a4** | **7** | internal | 135 | 135 | unavail. | 135 | 135 | absent | absent | major |
| **Atp2b1** | **34** | internal | 154 | 154 | unavail. | 154 | 154 | 154 | 166 | minor |
| **Atp2b1** | **35** | internal | unavail. | unavail. | unavail. | unavail. | unavail. | unavail. | unavail. | minor |
| **Atxn7l4** | **3** | 3' terminal | unavail. | 1296 | unavail. | unavail. | absent | absent | absent | major |
| **Atxn7l4** | **3a** | 3' terminal | 223 | 223 | unavail. | 223 | 223 | 223 | absent | major |
| **Bcas1** | **9** | internal | 42 | 42 | 42 | 42 | absent | absent | absent | major |
| **Bcas1** | **10** | internal | 168 | 165 | 168 | absent | absent | absent | absent | major |
| **Bcas1** | **11** | internal | 66 | 66 | 66 | absent | absent | absent | absent | major |
| **Brd9** | **5** | var5 | 116 | 116 | 116 | 116 | 116 | 119 | 122 | major |
| **Cacna1b** | **24a** | internal | 12 | 12 | 12 | 12 | 12 | 12 | 12 | major |
| **Camk2g** | **13a** | internal | 33 | 33 | 33 | 33 | 33 | 33 | 33 | minor |
| **Camk2g** | **13b** | internal | 69 | 69 | 66 | 69 | 69 | 69 | unavail. | minor |
| **Cask** | **19** | internal | 69 | 69 | 69 | 69 | 69 | 69 | 69 | major |
| **Catna2** | **17a** | internal | 144 | 144 | 144 | 144 | 144 | 147 | absent | major |
| **Ccdc64** | **8** | internal | 109 | 109 | 108 | unavail. | unavail. | unavail. | unavail. | minor |
| **Ccdc9** | **1a** | var3 | 105 | 105 | 114 | 105 | absent | 105 | 105 | minor |
| **Chl1** | **24** | internal | 159 | 162 | unavail. | 162 | 162 | unavail. | 162 | major |
| **Chl1** | **7a** | internal | 48 | 48 | unavail. | 48 | 48 | absent | absent | minor |
| **Clasp1** | **9** | internal | 117 | 117 | 117 | 117 | 117 | absent | 141 | minor |
| **Clstn1** | **10** | internal | 57 | 57 | 57 | 63 | 51 | absent | 51 | major |
| **Cp110** | **14** | internal | 60 | 60 | 60 | 60 | 60 | 60 | absent | major |
| **Cp110** | **14a** | internal | 86 | 86 | 86 | 86 | 83 | absent | absent | major |
| **Cp110** | **I14** | internal | 500 | 433 | 439 | absent | absent | absent | absent | minor |
| **Dab1** | **7b** | internal | 51 | 51 | 51 | 51 | absent | absent | unavail. | minor |
| **Dab1** | **7c** | internal | 48 | 48 | 48 | 51 | 57 | absent | unavail. | minor |
| **Ddr1** | **13a** | internal | 111 | 111 | 111 | 111 | unavail. | 102 | absent | major |
| **Dnajb5** | **1a** | internal | 145 | 145 | unavail. | unavail. | absent | absent | absent | minor |
| **Efna5** | **3a** | internal | 81 | 81 | 81 | 81 | 81 | 81 | 78 | major |
| **Epb4.1** | **13** | internal | 57 | 57 | 57 | 57 | 57 | absent | 59/75 | minor |
| **Epb4.1** | **14** | internal | 42 | 42 | 42 | 42 | 45 | 42 | 39 | minor |
| **Epb4.1L1** | **15** | internal | 87 | 87 | 87 | 87 | absent | absent | absent | major |
| **Epb4.1L1** | **16** | internal | 84 | 84 | 84 | 84 | 96 | 78 | absent | major |
| **Epb4.1L2** | **12a** | internal | 168 | 168 | unavail. | absent | absent | absent | absent | minor |
| **Epb4.1L2** | **12b** | internal | 54 | 54 | 57 | 54 | 54 | absent | absent | minor |
| **Epb4.1L3** | **16** | internal | 123 | 123 | 123 | 120 | 123 | 123 | absent | minor |
| **Epha5** | **4a** | internal | 66 | 66 | unavail. | 66 | 66 | absent | absent | minor |
| **Epim** | **10** | 3' terminal | 126 | 124 | 125 | unavail. | 128 | 129 | unavail. | major |
| **Falz** | **4** | internal | 162 | 162 | unavail. | 162 | 162 | 162 | unavail. | minor |
| **Gabrg2** | **9** | internal | 24 | 24 | 24 | 24 | 24 | 24 | 24 | major |
| **Glra2** | **3a** | internal | 68 | 68 | 68 | 68 | 68 | 68 | 68 | major |
| **Golga4** | **22a** | internal | absent | 134 | absent | absent | absent | absent | absent | minor |
| **Gphn** | **7a** | internal | 99 | 108 | unavail. | unavail. | 108 | absent | unavail. | major |
| **Gpr45** | **2** | 5' terminal | 207 | 219 | absent | absent | absent | absent | absent | major |
| **Gpr45** | **2a** | 5' terminal | unavail. | unavail. | unavail. | unavail. | absent | absent | absent | major |
| **Grik2** | **16** | internal | 87 | 87 | 87 | absent | absent | absent | absent | minor |
| **Grik2** | **16a** | internal | 132 | 139 | unavail. | unavail. | unavail. | absent | absent | minor |
| **Grin1** | **19** | internal | 111 | 111 | 111 | 111 | 111 | 111 | 111 | minor |
| **Idh3b** | **Intron 12** | 3' terminal | 115 | 118 | unavail. | 91 | unavail. | unavail. | unavail. | major |
| **Igsf4a** | **8** | internal | 84 | 84 | 90 | 69 | absent | absent | absent | major |
| **Igsf4b** | **2** | internal | 102 | 102 | 102 | absent | absent | absent | absent | minor |
| **ITGA3** |  | internal | 142 | 142 | 141 | 146 | absent | absent | 148 | major |
| **Kcnc2** | **11a** | internal | 104 | 104 | 104 | 104 | absent | absent | absent | minor |
| **Kcnma1** | **24a** | internal | 81 | 81 | 81 | 84 | 84 | 84 | 84 | minor |
| **Kcnq2** | **13** | internal | 30 | 30 | 30 | absent | 30 | absent | absent | minor |
| **Lrp1b** | **79** | internal | 99 | 99 | 99 | 99 | 99 | 99 | absent | major |
| **Lrrfip1** | **17** | terminal | unavail. | unavail. | unavail. | unavail. | unavail. | unavail. | unavail. | major |
| **Lrrfip1** | **17a** | terminal | 50 | 50 | 50 | 50 | 47 | 50 | 50 | major |
| **Map4** | **18** | internal | 190 | 190 | 190 | 172 | 178 | 172 | 181 | major |
| **Map4k4** | **22a** | internal | 192 | 192 | 192 | 192 | 192 | unavail. | absent | major |
| **Mapk9** | **7** | internal | 72 | 72 | 72 | 72 | 72 | 72 | 72 | major |
| **Mapk9** | **6a** | internal | 72 | 72 | 72 | 72 | 72 | 72 | 72 | minor |
| **Ncdn** | **1** | var3 | 35 | 35 | 35 | 35 | absent | absent | absent | major |
| **Neo1** | **27** | internal | 159 | 159 | 159 | 159 | 159 | 105 | 159 | major |
| **Nova1** | **4** | internal | 72 | 72 | 72 | 72 | 72 | 72 | absent | major |
| **Ntng1** | **5b** | internal | 66 | 66 | unavail. | 66 | 66 | absent | 45 | minor |
| **Pdlim5** | **6** | var5 | 419 | 419 | 419 | 413 | 416 | 449 | absent | major |
| **Plcb4** | **36a** | internal | 37 | 37 | 37 | 37 | 37 | 37 | 37 | major |
| **Plcl3** | **22a** | internal | 28 | 28 | 28 | 27 | 29 | 28 | 28 | minor |
| **Ptprf** | **19a** | internal | 33 | 33 | 33 | 33 | 33 | 33 | 33 | minor |
| **Ptprf** | **6a** | internal | 18 | 18 | 18 | 18 | 18 | 18 | 18 | minor |
| **Rap1ga1** | **27** | internal | 78 | 78 | 78 | absent | absent | 78 | 78 | minor |
| **Sh2bpsm1** | **9** | internal | 100 | 100 | absent | 100 | absent | absent | absent | major |
| **Skip** | **10** | internal | 87 | 87 | 87 | 87 | 87 | 90 | absent | major |
| **Smarcc2** | **16a** | internal | 93 | 93 | 93 | 93 | absent | absent | absent | minor |
| **Spna2** | **4** | internal | 60 | 60 | 60 | 60 | 60 | 60 | 60 | major |
| **Srr** | **2** | internal | 214 | 225 | 226 | absent | absent | absent | absent | major |
| **St7** | **7** | internal | 69 | 69 | 69 | absent | absent | absent | absent | minor |
| **Stxbp2** | **3** | internal | 82 | 82 | 82 | 82 | 82 | 82 | 82 | major |
| **Tacc2** | **10** | internal | 141 | 141 | 141 | 141 | 141 | 141 | 135 | minor |
| **Tpm3** | **6** | internal | 76 | 76 | 76 | 76 | 76 | 76 | 76 | major |
| **Tpm3** | **6a** | internal | 76 | 76 | 76 | 76 | 76 | 76 | 76 | minor |
