# Supplementary material for: Evolution of Nova-Dependent Splicing Regulation in the Brain
Source: PLoS Genet. 2007 Oct 12;3(10):e173. doi: 10.1371/journal.pgen.0030173 (PMC2014790; doi:10.1371/journal.pgen.0030173)
Supplement: Supplementary file 7 [file pgen.0030173.st005.doc]

Supplementary Table 5: Homology of orthologous alternative exons (% identity between the mouse exon and the orthologous exon).

|  |  | **% identity of orthologous exons to mouse alternative exons** | | | | | | |
| --- | --- | --- | --- | --- | --- | --- | --- | --- |
| **gene** | **exon** | **human** | **dog** | **rat** | **opossum** | **chicken** | **xenopus** | **zebrafish** |
| **Agrn** | **31a** | 83 | 96 | 100 | 96 | 83 | 67 | 88 |
| **Agrn** | **31b** | 91 | unavail. | unavail. | 64 | 55 | 39 | 48 |
| **Ank3** | **17** | unavail. | unavail. | unavail. | unavail. | unavail. | unavail. | unavail. |
| **Ank3** | **31** | 92 | 90 | 92 | 86 | 84 | 83 | 80 |
| **Ap1gbp1** | **6a** | 87 | 55 | 95 | 80 | absent | absent | absent |
| **Aplp2** | **12a** | 100 | 97 | 100 | 94 | 89 | 69 | 42 |
| **Arhgap21** | **8** | unavail. | unavail. | unavail. | unavail. | unavail. | unavail. | unavail. |
| **Atp13a4** | **7** | 87 | 88 | unavail. | 80 | 77 | absent | absent |
| **Atp2b1** | **34** | 99 | unavail. | 97 | 97 | 97 | 97 | 76 |
| **Atp2b1** | **35** | unavail. | unavail. | unavail. | unavail. | unavail. | unavail. | unavail. |
| **Atxn7l4** | **3** | unavail. | unavail. | unavail. | unavail. | unavail. | unavail. | unavail. |
| **Atxn7l4** | **3a** | 89 | unavail. | unavail. | 85 | 84 | 75 | absent |
| **Bcas1** | **9** | 81 | 81 | 95 | 81 | absent | absent | absent |
| **Bcas1** | **10** | 77 | unavail. | 91 | absent | absent | absent | absent |
| **Bcas1** | **11** | 79 | unavail. | 92 | absent | absent | absent | absent |
| **Brd9** | **5** | 92 | 87 | 90 | 87 | 87 | 63 | 57 |
| **Cacna1b** | **24a** | 92 | 100 | 100 | 92 | 92 | 67 | 100 |
| **Camk2g** | **13a** | 100 | 100 | 100 | 97 | 85 | 85 | 76 |
| **Camk2g** | **13b** | 99 | 99 | 94 | 96 | 78 | 74 | unavail. |
| **Cask** | **19** | 97 | 99 | 100 | 94 | 94 | 94 | 83 |
| **Catna2** | **17a** | 99 | 99 | 100 | 95 | 86 | 76 | absent |
| **Ccdc64** | **8** | 94 | unavail. | 94 | unavail. | unavail. | unavail. | unavail. |
| **Ccdc9** | **1a** | 93 | 89 | 56 | 82 | absent | 72 | 68 |
| **Chl1** | **24** | 90 | 90 | unavail. | 80 | 81 | unavail. | 60 |
| **Chl1** | **7a** | 92 | 90 | unavail. | 71 | 60 | absent | absent |
| **Clasp1** | **9** | 95 | 95 | 100 | 91 | 90 | absent | 61 |
| **Clstn1** | **10** | 84 | 77 | 93 | 73 | 60 | absent | 51 |
| **Cp110** | **14** | 100 | 98 | 100 | 85 | 85 | 82 | absent |
| **Cp110** | **14a** | 97 | 97 | 98 | 86 | 84 | absent | absent |
| **Cp110** | **I14** | unavail. | unavail. | unavail. | absent | absent | absent | absent |
| **Dab1** | **7b** | 73 | 69 | 94 | 69 | absent | absent | unavail. |
| **Dab1** | **7c** | 81 | 85 | 94 | 51 | 49 | absent | unavail. |
| **Ddr1** | **13a** | 99 | 97 | 100 | 92 | unavail. | 55 | absent |
| **Dnajb5** | **1a** | unavail. | unavail. | unavail. | unavail. | absent | absent | absent |
| **Efna5** | **3a** | 100 | 100 | 99 | 99 | 99 | 96 | 79 |
| **Epb4.1** | **13** | 93 | 96 | 95 | 86 | 75 | absent | 33 |
| **Epb4.1** | **14** | 93 | 93 | 98 | 83 | 80 | 64 | 48 |
| **Epb4.1L1** | **15** | 99 | 99 | 100 | 89 | absent | absent | absent |
| **Epb4.1L1** | **16** | 96 | 95 | 99 | 77 | 66 | 55 | absent |
| **Epb4.1L2** | **12a** | 86 | unavail. | unavail. | absent | absent | absent | absent |
| **Epb4.1L2** | **12b** | 87 | 83 | 82 | 76 | 69 | absent | absent |
| **Epb4.1L3** | **16** | 85 | 82 | 95 | 73 | 76 | 70 | absent |
| **Epha5** | **4a** | 98 | unavail. | unavail. | 94 | 88 | absent | absent |
| **Epim** | **10** | 77 | 72 | 98 | unavail. | 59 | 53 | unavail. |
| **Falz** | **4** | 96 | unavail. | unavail. | 93 | 84 | 71 | unavail. |
| **Gabrg2** | **9** | 100 | 100 | 100 | 100 | 100 | 92 | 75 |
| **Glra2** | **3a** | 99 | 99 | 99 | 97 | 99 | 96 | 79 |
| **Golga4** | **22a** | absent | absent | absent | absent | absent | absent | absent |
| **Gphn** | **7a** | 75 | unavail. | unavail. | unavail. | 82 | absent | unavail. |
| **Gpr45** | **2** | 76 | absent | absent | absent | absent | absent | absent |
| **Gpr45** | **2a** | unavail. | unavail. | unavail. | unavail. | absent | absent | absent |
| **Grik2** | **16** | 97 | 97 | 100 | absent | absent | absent | absent |
| **Grik2** | **16a** | 86 | 91 | unavail. | unavail. | unavail. | absent | absent |
| **Grin1** | **19** | 97 | 98 | 100 | 98 | 93 | 94 | 87 |
| **Idh3b** | **intron12** | 57 | unavail. | unavail. | 22 | unavail. | unavail. | unavail. |
| **Igsf4a** | **8** | 99 | 94 | 92 | 80 | absent | absent | absent |
| **Igsf4b** | **2** | 94 | 92 | 100 | absent | absent | absent | absent |
| **ITGA3** |  | 95 | 93 | 97 | 78 | absent | absent | 63 |
| **Kcnc2** | **11a** | 73 | 65 | 88 | 48 | absent | absent | absent |
| **Kcnma1** | **24a** | 95 | 94 | 100 | 85 | 83 | 81 | 80 |
| **Kcnq2** | **13** | 90 | 87 | 100 | absent | 80 | absent | absent |
| **Lrp1b** | **79** | 93 | 94 | 97 | 88 | 83 | 78 | absent |
| **Lrrfip1** | **17** | unavail. | unavail. | unavail. | unavail. | unavail. | unavail. | unavail. |
| **Lrrfip1** | **17a** | 86 | 86 | 94 | 78 | 64 | 68 | 66 |
| **Map4** | **18** | 92 | 93 | 99 | 65 | 45 | 41 | 39 |
| **Map4k4** | **22a** | 99 | 98 | 100 | 98 | 97 | unavail. | absent |
| **Mapk9** | **7** | 99 | 97 | 100 | 94 | 93 | 79 | 79 |
| **Mapk9** | **6a** | 99 | 99 | 100 | 94 | 93 | 82 | 81 |
| **Ncdn** | **1** | 100 | 100 | 100 | 91 | absent | absent | absent |
| **Neo1** | **27** | 98 | 96 | 99 | 91 | 89 | 42 | 77 |
| **Nova1** | **4** | 97 | 97 | 100 | 99 | 99 | 86 | absent |
| **Ntng1** | **5b** | 98 | unavail. | unavail. | 92 | 92 | absent | 52 |
| **Pdlim5** | **6** | unavail. | unavail. | unavail. | unavail. | unavail. | unavail. | absent |
| **Plcb4** | **36a** | 97 | 97 | 95 | 95 | 92 | 81 | 65 |
| **Plcl3** | **22a** | 82 | 82 | 93 | 89 | 83 | 75 | 64 |
| **Ptprf** | **19a** | 94 | 97 | 100 | 91 | 94 | 94 | 70 |
| **Ptprf** | **6a** | 100 | 100 | 100 | 83 | 89 | 94 | 72 |
| **Rap1ga1** | **27** | 92 | 94 | 100 | absent | absent | 76 | 76 |
| **Sh2bpsm1** | **9** | 93 | 96 | absent | 85 | absent | absent | absent |
| **Skip** | **10** | 95 | 95 | 99 | 91 | 86 | 53 | absent |
| **Smarcc2** | **16a** | 98 | 98 | 98 | 90 | absent | absent | absent |
| **Spna2** | **4** | 98 | 95 | 100 | 93 | 90 | 82 | 72 |
| **Srr** | **2** | 75 | unavail. | 94 | absent | absent | absent | absent |
| **St7** | **7** | 100 | 100 | 100 | absent | absent | absent | absent |
| **Stxbp2** | **3** | 90 | 83 | 96 | 83 | 76 | 72 | 73 |
| **Tacc2** | **10** | 89 | 90 | 96 | 79 | 74 | 63 | 51 |
| **Tpm3** | **6** | 97 | 99 | 96 | 96 | 82 | 72 | 62 |
| **Tpm3** | **6a** | 100 | 100 | 99 | 99 | 96 | 87 | 83 |
