# Supplementary material for: Evolution of Nova-Dependent Splicing Regulation in the Brain
Source: PLoS Genet. 2007 Oct 12;3(10):e173. doi: 10.1371/journal.pgen.0030173 (PMC2014790; doi:10.1371/journal.pgen.0030173)
Supplement: Supplementary file 8 [file pgen.0030173.st006.doc]

PCR primer list

| Agrn_chick_f:CCACAATGCCGTGACAAAGAG | Agrn_chick_r:GCCCCTTCCCACTCCACAAG |
| --- | --- |
| Agrn_fish_f:GCTCCAAAGACGTTTGTGAGAATG | Agrn_fish_r:TTTATTCACCAGCAGGGCTTTC |
| Ank3_chick_f:GAGGATGCAATGACAGGTGACAC | Ank3_chick_r:GAATCAAACTCCCTCTGAAACGTC |
| Aplp2_chick_f:GCAAACCTTTCCGTCCGTTC | Aplp2_chick_r:GTCAGACAGCCTATCCAACTCAGTC |
| Aplp2_fish_f:GTGTTGAGGTTCCTGCTTTTGAAG | Aplp2_fish_r:CTCCAATCAGACCATCCAGACTG |
| Brd9_chick_f:ACACACAGGCTTTAAGATGATGAGC | Brd9_chick_r:CGATTGATCCTATCCCGAGCTTC |
| Brd9_fish_f:AAGGCTGCTAAGAAACTCCTCCAC | Brd9_fish_r:CTGTGCTGTCCGTCAAACTGC |
| Cacna1b_chick_f:GCTCCTATTTCCGTGACCTGTG | Cacna1b_chick_r:TTGATGTCCTTGCCTTTGGTTC |
| Cacna1b_fish_f:TTGGACTTCATTGTGGTCAGTGG | Cacna1b_fish_r:TTCAGAGGTCGCAAGACTCGCAAC |
| Camk2g_chick_f:GGTGCAATCCTCACAACGATG | Camk26_chick_r:TGGACAACAGTTGTTTGAGGCTC |
| Camk2g_fish_f:CTGAACAAAAAGTCTGATGGCGTG | Camk2g_fish_r:TTGGTGCTGCTGCTTACTACACTG |
| Clasp1_chick_f:GCACTCCAGCAGCAGAACCAG | Clasp1_chick_r:TTCATTCAGGTCCTCTTGACTACGG |
| Clstn1_chick_f:TATCCGCTTCATCCTTCAAAAATAG | Clstn1_chick_r:AAACCTGCCAGATTGCCTCG |
| Dab1_chick_f:CCAGCCAAAAGAAGGAAGGTG | Dab1_chick_r:GAGGTTACATCGGGAGGAGTGG |
| Efna_chick_f:CTGCGATCCCAGATAATGGAAG | Efna_chick_r:GGATGGCTCGGCTGACTCAC |
| Efna_fish_f:CGGCAGAAGGTCATGTCTCAAGC | Efna_fish_r:GCGAGATGCAGAGCAGCAGGAG |
| Epb4.1_chick_f:GCCCTCGCTGCATCAAAAGTAG | Epb4.1_chick_r:TCCAACATTAAATTGCTATGCCTG |
| Epb4.1_fish_f:GAAGAGTGAGGTGAGGGCAG | Epb4.1_fish_r:TCTGTCTGTGTCTTATCCACGTC |
| EPB41L2_chck_f:AAAGCGGCGTATCACACCTCTGTC | EPB41L2_chick_r:TTCCCCAGCATCAGTGGCAACTTG |
| Golga4_chick_f:CGTCCCTCTCTTTGGAGAGCC | Golga4_chick_r:GCTGGGAATTTCAGGACTGTTG |
| Gphn_chick_f:TCACAAGCAGACAGAGGACAAAGG | Gphn_chick_r:CCTGAACACCACGAGAAATGATGG |
| Kcnma1_chick_f:TTACAACAGGAGCCAACATCCC | Kcnma1_chick_r:GACAGCAAATGCGGTTCCAC |
| Kcnma1_fish_f:GGTCGTCATCGTCGTCTTGG | Kcnma1_fish_r:GGTCATCTCCTGATAACAGTCCCG |
| Lrrfip_chick_f:GACTCTGACATAGCCACCAATGG | Lrrfip_chick_r:AGGACTCTCGTTCGCCAATGAG |
| Lrrfip_chick_r2:AGACTCCTCTTTGTGTTCCTCACG | Map4_chick_f:CCAAAGTGGGTTCTCTGGACAAC |
| Map4_chick_r:GCCTTCGCCTTCTCACGG | Map4_fish_r:AAAATGGGATCAATGGACAATGTAG |
| Map4_fish_f:CGGAAGTTCAGCTTGTGGGACTC | Map4k4_chick_f:AGTGAACCTGCCTTGACTCCTTC |
| Map4k4_chick_r:TCGCTCTGTGGTCTTGTGTTTG | Neo1_chick_f:CCCAAGATGATGATGCCTTTTG |
| Neo1_chick_r:TGAGCACTGCCCAGGTAAGC | Neo1_fish_f:TGATGCCCTTTGATGCACAAC |
| Neo1_fish_r:GAGCTGTGAAAGCTAATGTCCGTC | Plcl3_chick_f:CTTCTATGCCAGTGGACAAATTCC |
| Plcl3_chick_r:CCTTCTCAGTTTTATCGCCATCAC | Plcl3_fish_f:CTGGGATAAACTCCTGCACAGTAAC |
| Plcl3_fish_r:TGAAATGGATCTGGGCTTCTCAC | Ptprf_19a_chick_f:AGGCGGCTCAACTACCAGACTC |
| Ptprf_19a_chick_r:ATGTTTGTCGGCGAGGTCGGTCACG | Ptprf_chick_f:ATGCTCTGTGCTGCCAGTGGTAAC |
| Ptprf_chick_r:TATTTGCCCTTGGTCCGACTCCTC | Ptprf_fish_f:ACTTGCCCTGGTCTGATTCCTC |
| Ptprf_fish_r:AACAACTACAATGCTCTGTGCTGC | St7_chick_f:GCAAAAAGCCTGGAGAGAGAGAAAC |
| St7_chick_r:AGCCTTCAGAGCTTGCTTGAACAG | St7_fish_f:CATGAAGCTCTGGATCTGGAGG |
| St7_fish_r:AGGAGAATATACGCCGTCGCAC | Stxbp2_chick_f:AAGAAGGTGAAGAAGAAGGGCG |
| Stxbp2_chick_r:GTGATGAGATAGACAGCCTCCAGG | Stxbp2_fish_f:TCAAGAGTGTGAAGAAAGATGGGG |
| Stxbp2_fish_r:TTCGTTTGCTGATGTCCTCCAC | Tacc2_chick_f:CAAAACAGAAGGACTTAGAGCCC |
| Tacc2_chick_r:CTGGGGGTAGAGAAGACTCGTGG |  |
